# Life-history trade-offs explain local adaptation in *Arabidopsis thaliana*

**DOI:** 10.1101/2025.05.18.654693

**Published:** 2025-05-23

**Authors:** Benjamin Brachi, Danièle L. Filiault, Rahul Pisupati, Tal Dahan-Meir, Anna Igolkina, Alison Anastasio, Mathew S. Box, Susan Duncan, Talia L. Karasov, Envel Kerdaffrec, Laura Merwin, Timothy C. Morton, Viktoria Nizhynska, Polina Yu. Novikova, Fernando Rabanal, Takashi Tsuchimatsu, Torbjörn Säll, Caroline Dean, Svante Holm, Joy Bergelson, Magnus Nordborg

**Affiliations:** 1 Department of Ecology and Evolution, University of Chicago, Chicago, Illinois, USA; 2 University of Bordeaux, INRAE, BIOGECO, Cestas, France; 3 Gregor Mendel Institute, Austrian Academy of Sciences, Vienna BioCenter, Vienna, Austria; 4 John Innes Center, Norwich, United Kingdom; 5 Department of Molecular Cell Biology, Lund University, Lund, Sweden; 6 Department of Natural Sciences, Mid-Sweden University, Sundsvall, Sweden; 7 Department of Biology, New York University, New York, USA

## Abstract

Local adaptation has been demonstrated in many organisms, but the traits involved, and the temporal and spatial scales at which selection acts are generally unknown. We carried out a multi-year study of 200 accessions (natural inbred lines) of Swedish *Arabidopsis thaliana* using local field sites and a combination of common-garden experiments that measured adult survival and fecundity, and selection experiments that measured fitness over the full life cycle. We found evidence of strong and variable selection, with particular genotypes favored more than five-fold in certain years and locations. Fecundity showed evidence of classical local adaptation, with accessions generally performing better close to their home. However, southern accessions usually had the highest fecundity—but were far more sensitive to harsh winters and slug herbivory, which strongly decreased both survival and fecundity. Accessions originally sampled on beaches had low fecundity in all environments, but massively outperformed all other accessions in the selection experiments, presumably due to an advantage during seedling establishment associated with their very large seeds. We conclude that local adaptation in *A. thaliana* reflects strong temporally and spatially varying selection on multiple traits, generally involving trade-offs and different life-history strategies, making fitness difficult to predict and measure.

## Introduction

Species reside in a heterogeneous landscape, with selective forces varying across time and space. When these selective forces are sufficiently consistent and spatially restricted, they may result in local adaptation whereby genotypes perform better at home than away (reviewed in Savolainen et al., 2013). The first demonstrations of such adaptive differentiation among populations focused on plants, due in part to the ease of reciprocal transplant "common-garden" experiments (Turesson, 1922; Clausen et al., 1940). This tradition has continued in plant biology until the present day (Sork, 2017). Reciprocal transplant studies typically involve either phenotypically differentiated ecotypes (Turesson, 1922), presumably resulting from strong selection, or geographically disparate accessions that have a long history of evolving independently. But selection plays out in a dynamical landscape with changing selective pressures, interbreeding genotypes, and genetic variation that is restricted to the regional pool. Within this more realistic context, we know very little about the relevant spatial and temporal scales at which selection operates, and how short-term selection on various traits combine across time and space to shape long-term patterns of evolution.

At the genetic level, the picture is similarly murky, due in part to issues of scale. Even seemingly homogeneous fields vary on a micro-scale, with impacts on the relative fitness of plant genotypes (Stratton, 1994, 1995). Such heterogeneity forms a mosaic that jointly determines evolution locally; for example Frachon et al. (2017) studied rapid evolution in a natural populations of *A. thaliana* and found that SNPs associated with intermediate levels of pleiotropy, not only across traits but across micro-environments, were those that changed in frequency. Results like these suggest a rich complexity in how short-term selection combines across time and space.

The present study sought to gain insight into local adaptation in *A. thaliana*. While not the first study on local adaptation in *A. thaliana*, past work, including our own, had involved very sparse spatial sampling—either ≈ 150 globally sampled accessions (Hancock et al., 2011; Fournier-Level et al., 2011) or a RIL population formed by crossing an Italian and a northern Swedish accession (Ågren and Schemske, 2012)—and we were concerned that fitness differences observed on this scale would either be impossible to interpret, or be dominated by well-known phenomena, such as the connection between flowering time and latitude. We were also worried that the traditional common-garden experiments used did not consider selection during seedling establishment, which we suspected would play a major role in an annual, weedy species like *A. thaliana*.

**Figure 1.**
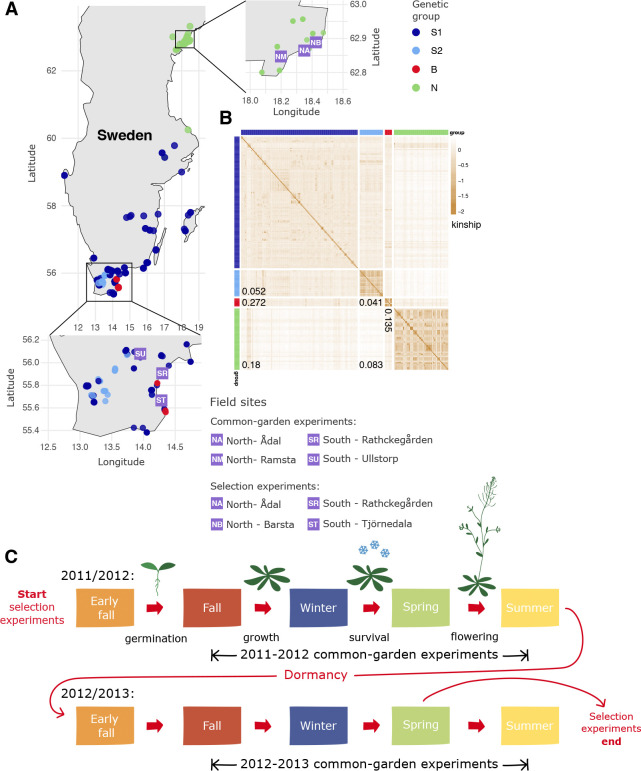
Experimental set-up. (**A**) Location of the 6 field sites and origin of the 200 accessions. (**B**) Heat-map showing kinship between the accessions, hierarchically clustered by similarity. Marginal colors indicate membership in one of four genetic groups—the same colors are used for the sampling locations in panel A. Numbers are $F_{ST}$ estimates between groups. (**C**) Schematic of the experiments. In the common-garden experiments, which were replicated over two consecutive seasons, established seedlings (3 blocks of 8 replicates of 200 accessions per experiment) were transplanted into the field in fall, and overwinter survival and fecundity assessed the following spring. In the evolution experiments, seeds were sown in equal numbers in early fall 2011 and surviving descendants sequenced before flowering in 2013.

For these reasons, we decided to carry out a study in Sweden, using 200 Swedish accessions, most of which we had collected ourselves and whose habitats we were therefore familiar with (Figure 1). The study focused on differences between the High Coast region in northern Sweden, where *A. thaliana* is mostly found on naturally eroded, south-facing slopes, and the heavily agricultural Skåne region in southern Sweden, where the species can be found in a variety of habitats, most of them associated with human disturbance. We additionally added one field site on a beach in eastern Skåne, as this habitat stood out as an unusual habitat with a very large stable population. In an attempt to include seedling establishment, we complemented standard common-garden experiments with novel selection experiments in which we sowed equal numbers of seeds of each accession in suitable habitats hoping to estimate fitness as presence in later generations—although it is important to note that when the study was started 15 years ago, we had no idea when it would be economically feasible to genotype any survivors.

## Results

### Characterizing population structure

Although preliminary data had suggested clear genetic differentiation between northern and southern Sweden (Nordborg et al., 2005; Atwell et al., 2010), the full extent did not become clear until genome-wide SNP data from larger numbers of accessions became available (Long et al., 2013; Huber et al., 2014; 1001 Genomes Consortium, 2016). To describe the structure of the sample used here, we let ADMIXTURE (Alexander et al., 2009) divide the 200 accessions into distinct (supposedly random-mating) groups. As with all clustering methods, the interpretation is quite arbitrary: we found that four groups provided a good fit to the geographic origin of the accessions and the overall structure of the sampling (Figure 1, Figure 1—figure Supplement 1). All northern accessions fell into one group, N; S2 consists exclusively of accessions from Skåne (Sweden’s southern-most province), with 60% being collected in urban areas; and B consists solely of accessions collected directly on Baltic Sea beaches in southern Sweden. The more heterogeneous S1 group is predominantly southern, but includes many isolated accessions from central Sweden. The divergence between these groups is substantial, as can be seen from the $F_{ST}$ values (Figure 1; see also the correlation in SNP allele frequency between groups in Figure 1—figure Supplement 2).

### Survival conditional on establishment in common gardens

**Figure 2.**
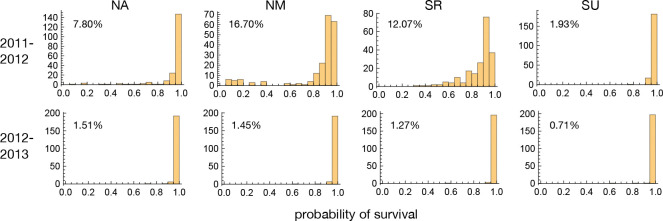
The distribution of accession survival probabilities across sites and years. Numbers in plots give total mortality in each experiment.

Overwinter survival varied greatly between sites and years. In 2012–13, overall mortality was less than 2% in all four sites, and there was effectively no meaningful variation for survival (Figure 2). The same was true for one of the southern sites in 2011–12, SU, but the remaining three sites saw substantial mortality, allowing us to compare accessions and groups.

**Figure 3.**
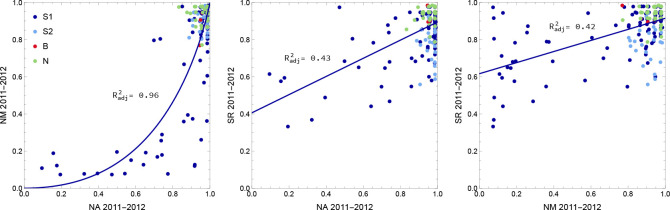
The joint distribution of accession survival probabilities between the three experiments with significant mortality. The curves were fitted using the S1 accessions only.

In these three experiments, southern accessions experienced dramatically increased mortality. In the two northern sites, NA and NM, where mortality was over 50% for many accessions, members of the S1 group were particularly affected, and our results suggest that conditions were more severe in NM both because overall mortality was twice as high (Figure 2) and because accessions with high mortality in NA always had high mortality in NM, whereas the converse was not true (Figure 3). Supporting this, a standard liability-threshold model (see Methods) with higher exposure in NM fits the data well.

Occasionally reduced overwinter survival of southern accessions in northern sites is not surprising and suggests lack of adaptation to colder winters. It is consistent with the results of Oakley et al. (2023), who, using a cross between an Italian and a northern Swedish accession (Ågren and Schemske, 2012), observed that Italian genotypes survived northern winters less well than northern Swedish genotypes in harsher years—whereas there was no difference in milder years.

**Figure 4.**
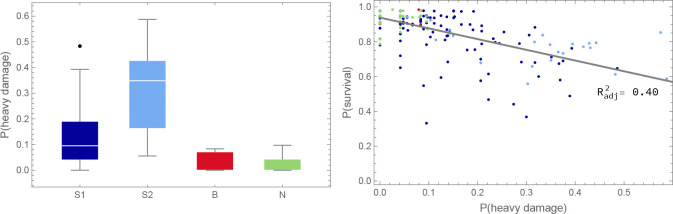
Overwinter survival was affected by herbivory. Left: Slug damage in the SR 2011–12 experiment affected groups differently (Kruskal-Wallis test: p<0.01 for all comparisons). Right: Average slug damage for an accession decreased its probability of survival (ANOVA: $p=1.6\times 10^{-23}$).

In contrast, higher mortality of southern accessions in the southern SR site in 2011–12 was not expected. While only S1 accessions experienced higher mortality in the two northern sites, S2 accessions were also strongly affected in the SR experiment, suggesting a different cause of mortality (Figure 3, Figure 2—figure Supplement 1). As it turns out, a major cause was observed: herbivory. The 2011–12 SR experiment was attacked by slugs, which caused substantial damage to leaves in the fall. We scored this damage and found that it both predicted overwinter survival and varied substantially between accessions, with S1 and S2 accessions being far more susceptible than B and or N accessions (Figure 4). Interestingly, despite the different causes, mortality in the SR experiment was correlated with mortality in the north for S1 accessions (Figure 3). In other words, the same accessions tended to die in both. This could reflect a direct causal connection, *e.g.* slug resistance also protects against cold. However, an indirect association is likely. Supporting this notion, survival in the SR experiment remained correlated with survival in the northern experiments after correcting for slug damage (Figure 4—figure Supplement 1).

**Figure 5.**
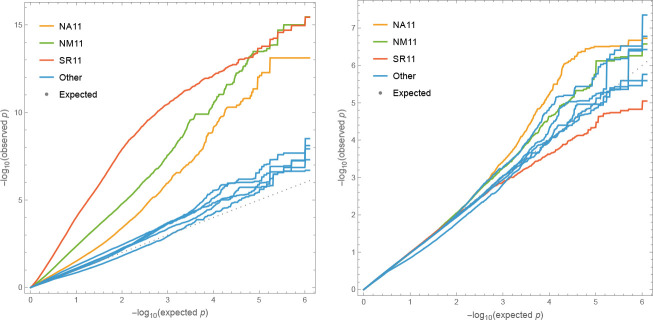
Quantile-quantile plots of p-values for overwinter survival GWAS against the expected uniform distribution. Left plot without correction for structure; right plot including a standard mixed-linear model kinship correction. Only SNPs with Minor Allele Frequency (MAF) greater than 5% were included to avoid outlier affects. Experiments with significant mortality exhibit genome-wide inflation of significance, while remaining experiments demonstrate that tails are inflated even for noise phenotypes.

GWAS for overwinter survival exhibit strong population structure confounding (Figure 5), which is precisely what is expected for traits that have been selected to differ between genetically differentiated groups (Yu et al., 2006; Zhao et al., 2007; Atwell et al., 2010; ***Platt et al., 2010b***). Standard statistical approaches for addressing this problem, like kinship-corrected mixed-linear models, have a tendency to over-correct, effectively "throwing out the baby with the bath water". In light of this, drawing conclusions from GWAS results alone, without supporting experiments, is not warranted. That said, we note that one of the top association peaks for survival in the SR 2011–12 experiment (Figure 5—figure Supplement 3) includes the *AOP* cluster, which has been shown to play a major role in natural variation for glucosinolate profiles and defense against herbivory, even on local scales (Kliebenstein et al., 2001; Gloss et al., 2022). The peak appears to involve a haplotype over 30 kb in length, which is consistent with a history of strong selection on this locus (Kliebenstein et al., 2001; Sasaki et al., 2021). We find no association at the even more important *MAM* cluster, consistent with this locus not being variable in Sweden (Katz et al., 2021). This suggests that the variation for slug damage seen in Figure 4 may be partly mediated by glucosinolate production.

In general, far less work has been done on overwinter survival than on traits like flowering time or glucosinolate profiles, and there are therefore fewer *a priori* candidates to help interpret GWAS results. A possible exception is *SVP*, which is under one of the strongest (uncorrected) association peaks in NM 2011–12 (Figure 5—figure Supplement 4). This gene encodes a MADS-box transcription factor that plays a major role in flowering-time variation (Méndez-Vigo et al., 2013; Guo et al., 2023) via thermo-sensory pathways (Lee et al., 2013), and hence may also be involved in preparing plants for winter. *SVP* is frequently identified in selection scans because it exhibits extremely high population differentiation—consistent with a role in local adaptation (Horton et al., 2012; Zou et al., 2017; Guo et al., 2023). However, precisely because of this, *SVP* will be associated with any trait that shows geographic differentiation and further experiments will be required to determine which adaptive traits it actually influences.

Interestingly, GWAS for overwinter survival in the north in 2011–12 finds no association at *CBF2*, which has been shown to play a role in freezing tolerance in the above-mentioned cross between a northern Swedish and an Italian accession due to a loss-of-function allele in the Italian parent (Gehan et al., 2015; Lee et al., 2024). We used the BLAST-based simsearch tool from the Pannagram package (Igolkina et al., 2025) to look for the causal 13-bp deletion in close to 600 independently assembled *A. thaliana* genomes, including over 150 from Sweden (The 1001G+ Consortium, 2024), but found the deletion only in the Italian accession used as RIL parent by Ågren and Schemske (2012). This suggest that the loss-of-function allele identified by Gehan et al. (2015) is rare and does not play a major role in the genetic architecture of freezing tolerance in Sweden (or Europe-wide). Thus, although our results are consistent with those of Oakley et al. (2023) at the phenotypic level, the genetic basis appears to be different.

### Fecundity conditional on survival in common gardens

**Figure 6.**
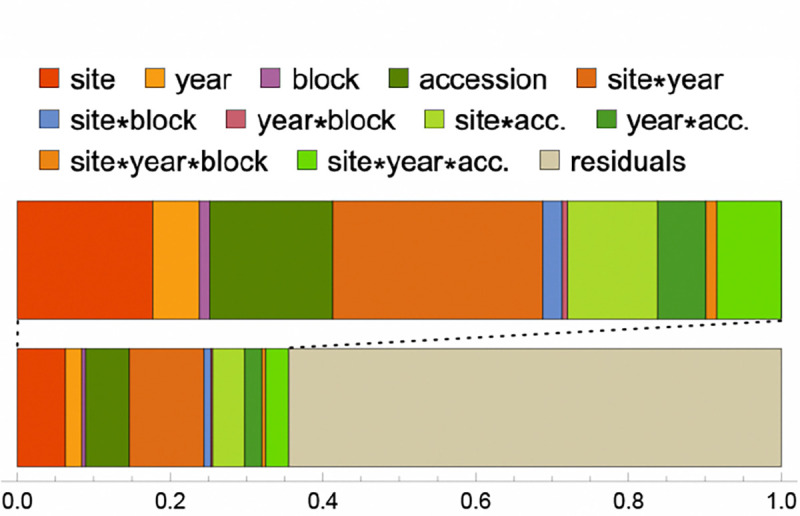
Variance-partitioning (ANOVA) from full model of fecundity.

Fecundity was estimated from photos of harvested mature plants, thus also capturing the amount of biomass these annual plants invested in reproduction (see Brachi et al., 2022, for details). Linear modeling of the entire study (*i.e.*, including all 8 experiments) showed that while the *accession*, *year*, and *site* terms were all highly significant, a greater proportion of explainable variation was due to interactions between them (Figure 6). In particular, interactions involving *accession* indicate that the genetic control of fecundity varied between experiments, a prerequisite for local adaptation. Significant year*site and year*site*accession interactions demonstrate that fecundity varies from year-to-year, with this effect differing among sites.

**Figure 7.**
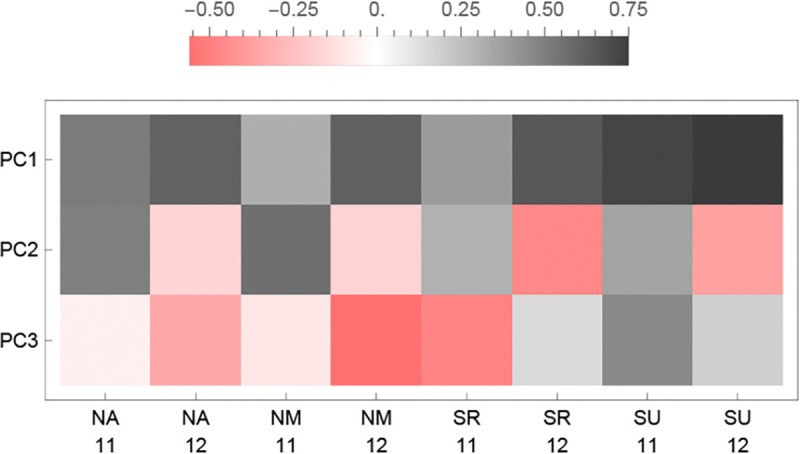
The loadings of the first three PCs from a PCA of the fecundity BLUPs on the eight experiments.

In order to compare the performance of accessions, we used standard mixed-effects models to estimate the accession-effect on fecundity in each experiment (*i.e.*, BLUPs; see Methods), and identified patterns across the experiments using Principal Components Analysis (Figure 7). The first principal component (PC1; explaining 38% of the variance) identifies a common pattern across experiments; the second component (PC2; 15% of the variance) switches sign between the years of the study; and the third (PC3; 13% of the variance) switches sign between northern and southern experiments—except that SR 2011–2012, the experiment with heavy overwinter mortality due to slugs, behaves like a northern experiment.

**Figure 8.**
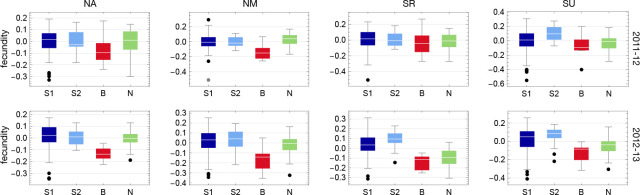
The distribution of fecundity BLUPs for each experiment by group.

These patterns are consistent with the large interaction effects seen in Figure 6, and the full distribution of BLUPs reveals what is causing them (Figure 8). PC1 reflects the fact that S1 and S2 accessions generally have higher fecundity than B and N accessions. The B accessions have the lowest median fecundity in all eight experiments (significantly so in only 5 out of 8, but note that the probability of any one group consistently being last is ${\left(1/4\right)}^{7}=6\times 10^{-5}$ and the N accessions have the second lowest fecundity in all southern experiments and all 2012–13 experiments (significantly lower than S1 and S2 in 4 out of 6 experiments). Only in the northern experiments in 2011–12 was this trend broken, although the differences are small and not statistically significant.

**Figure 9.**
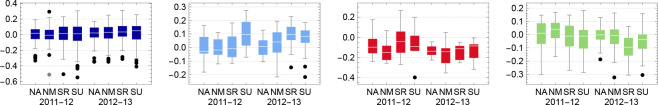
The distribution of fecundity BLUPs for each group by year and site (*cf.* Figure 8).

The pattern captured by PC1 is far stronger in 2012–13 than in 2011–12, and this is captured by PC2. As can be seen in Figure 9, S1 and S2 accessions generally had higher fecundity in 2012–13 than in 2011–12, while the reverse was true for B and N accessions.

PC3, finally, captures the fact that, within years, southern accessions (S1, S2, and B) generally perform better in southern than in northern experiments, while the opposite is true for the northern accessions (N). The probability of this pattern in a particular experiment is 1/6, and it occurs in 7 of the 8 experiments (one-tailed probability 2.4 × 10^−5^).

In the three experiments where high mortality was observed, fecundity was correlated with overwinter survival, suggesting that surviving members of affected genotypes were weakened by whatever caused death among their (inbred) siblings (Figure 8—figure Supplement 1). This effect thus decreased the fecundity of the S1 accessions in NA and NM 2011–12, and of the S1 and S2 accessions in SR 2011–12, presumably explaining why this latter experiment looks "northern" in the PCA (Figure 7).

**Figure 10.**
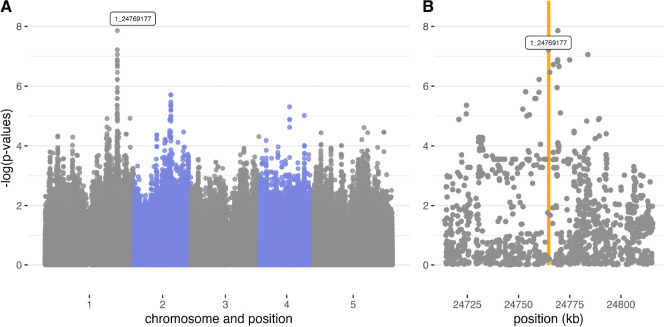
GWAS of rosette purpleness in SU 2011–12 identifying *Production of Anthocyanin Pigment 2, (PAP2, AT1G66390)*, located on chromosome 1 between 24,763,941 and 24,765,541 bp. A. Genomen-wide Manhattan plot B. Zoom-in on the 100 kb window around *PAP2*, represented by the orange bar.

In addition to fecundity, we also noticed variation in the extent to which leaves had turned purple in the fall, presumably as a sign of stress. We scored this from photos (see Rosette purpleness in Methods). Differences between accessions explained 4–26.6% of color variation across the six experiments in which it was called, and correlations with mortality and fecundity were mostly insignificant. Interestingly, having a purple rosette appeared to have a relatively simple genetic basis: in the experiment where accession explained the most variation, GWAS revealed single major locus in the anthocyanin production pathway, *PAP2* (Figure 10). The 10 top SNPs, all located between 24,764,623 and 24,783,821 bp on chromosome 1, jointly explained 17.7% of genetic variation among accessions.

**Figure 11.**
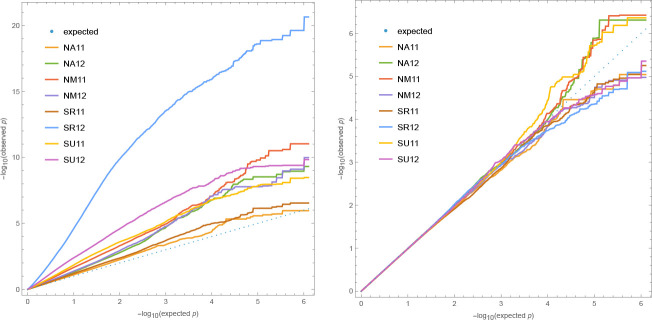
Quantile-quantile plots for GWAS fecundity data. Left plot without correction for structure; right plot including a standard mixed-linear model kinship correction. The extreme confounding for SR 12 reflects the unusually large difference between S1/S2 and B/N in this experiment (Figure 8). Only SNPs with Minor Allele Frequency (MAF) greater than 5% were included to avoid outlier affects.

In contrast, fecundity is obviously a complex trait. It is downstream of overwinter survival (in itself a complex trait), and it is characterized by complex genotype-by-environment interactions. In addition, it is strongly correlated with population structure. As expected, GWAS results thus exhibit strong population-structure confounding, and while attempting to eliminate this confounding statistically eliminates false positives, it also removes signal (Figure 11).

That said, the GWAS identify many clear peaks of association that are suggestive of large-effect polymorphisms. Some of these are shared between experiments, consistent with the patterns discussed above (*e.g.*, the dominant S1/S2 vs B/N patterns identified by PC1 in Figure 7). Each peak includes on the order of ten genes, but in the absence of strong priors for which genes might affect a highly complex trait like fecundity, speculating about causality is futile. These results will hopefully guide future experiments.

### Fitness in selection experiments

#### Estimating fitness

Our experimental evolution experiments were also carried out at four sites, two in the north, and two in the south, with one site in each region associated with a common garden experiment (Figure 1). One of the southern sites, ST, was located on a beach, with members of the B group as its local population. Although native plants growing nearby, the sites were assessed to be free of *A. thaliana*, but were deemed to be suitable habitats. We were correct about the second part, but not the first, as we shall see below.

At each site, seeds from the 200 accessions were mixed in equal numbers and sown at relatively high densities to establish three to five independent experimental plots, which were then allowed to complete a full life cycle under natural conditions (including seed dormancy, seedling establishment, and competition). At least 70 plants per plot (1,238 plants in total) were randomly sampled after the second winter. These plants were individually genotyped using low-coverage short-read sequencing to assess the composition of each experimental population. Given that all accessions started the experiment at equal frequency, fitness for each accession in each site was simply calculated using its sampled population frequency at that site.

Genotyping turned out to be challenging, mostly because of the difficulties involved in extracting DNA from plants that were collected under field conditions and which were often tiny and half-dead. As described in Methods (see Figure 19), we concluded that, of 1,174 genotyped samples: 75.4% were homozygous individuals matching our 200 experimental accessions, 15.8% were homozygous individuals that did not match our experimental accessions (*i.e.*, they were native “volunteers"), and 8.8% were extensively heterozygous (either due to recent outcrossing or sample contamination). The native volunteers were found in every experiment (Figure 19—figure Supplement 1)—thus demonstrating that we did indeed pick sites suitable for *A. thaliana*—and they do not pose a problem, as they do not affect the relative fitness estimates for the experimental accessions.

However, the presence of *bona fide* natives alerted us to the fact that our fitness estimates could be confounded by the inclusion of natives indistinguishable from our experimental accessions. To investigate this we used background knowledge of the population structure in *A. thaliana* to identify experimental accessions that had originally been collected close enough to an experimental site for it to be plausible that nearly identical plants could be native to that site (see Methods). We found that this problem could potentially be serious in two of the sites: NB, where 22% of putatively experimental samples were potentially natives, and ST, where the proportion of potential natives was 52%. The proportion of potential natives at NA was only 3% and the SR site could not have been affected as no experimental accession had been collected nearby.

**Figure 12.**
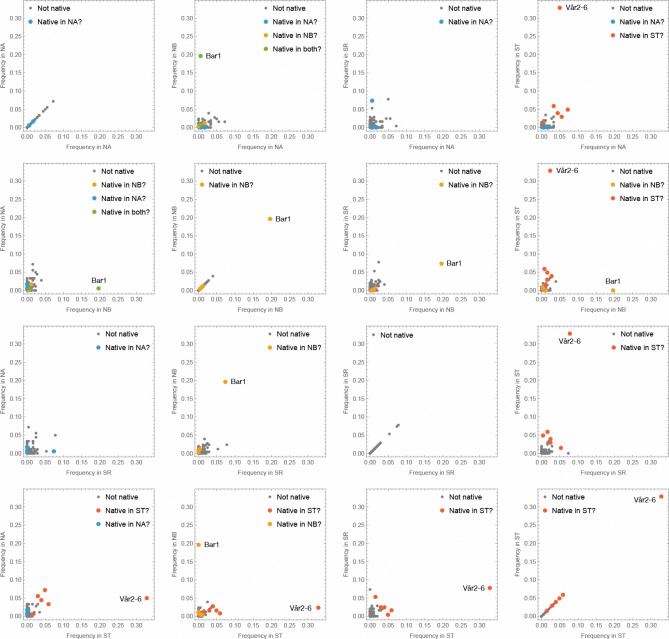
Scatter-plots comparing estimated accession frequencies in the four selection experiments. Accessions that are potential natives in a site are indicated by color and the two obvious outliers (Bar1 in NB, and Vår2–6 in ST) are highlighted.

As it turns out, two accessions make up most of these potential natives and are also obvious outliers in the expected experimental populations (Figure 12). At the NB (Barsta) site, the accession Bar1 (originally sampled in Barsta) comprised 20% of sampled individuals (and 88% of potential natives). While it is theoretically possible that Bar1 is extremely well-adapted to its local habitat and thus outperformed all other accessions, increasing 40-fold in frequency during the course of the experiment (all accessions started the experiment at frequency 0.5%), it seems far more likely that most of the Bar1 individuals sampled at NB hail from the native, non-experimental population.

Similarly, at the ST (beach) site, the accession Vår2–6, originally collected about 3.5 km further south along the same beach, comprised 33% of sampled individuals, an implied 65-fold increased in frequency. Removing these two accessions from the study eliminates 69% of all potential natives, effectively eliminating the problem at all sites except for ST, which still has 28% potential natives. This reflects the facts that all experimental accessions hailing from the beach with the ST site are potential natives, *and* that these accessions were over-represented in the individuals sampled in the ST experiment—*prima facie* consistent both with higher fitness of beach accessions and confounding by the native, non-experimental population. However, looking across experimental sites (Figure 12, Figure 12—figure Supplement 1), we see that these accessions were over-represented in *all* experiments, including at the three sites where their numbers could not have been inflated by native contributions, and we therefore conclude that they actually had higher fitness and treat them as experimental samples. As we shall see below, other observations support this conclusion. Nevertheless, in the ST site, the fitness estimates maybe be inflated by the inclusion of natives, but none of our conclusions depend on this.

Importantly, the eliminated beach accession, Vår2–6, also had very high fitness in the other experiments (5.0% in NA, 2.4% in NB, 7.8% in SR) suggesting that its extraordinarily high estimated fitness in its native habitat (ST) probably was not *only* due to confounding by natives, but also reflect general fitness advantage plus local adaptation. This, of course, makes its exclusion conservative. Note that the same is not true for the Bar1 accession (0.6% in NA [where it was also potentially native], 7.3% in SR, 0% in ST).

#### Comparing accessions and groups

**Figure 13.**
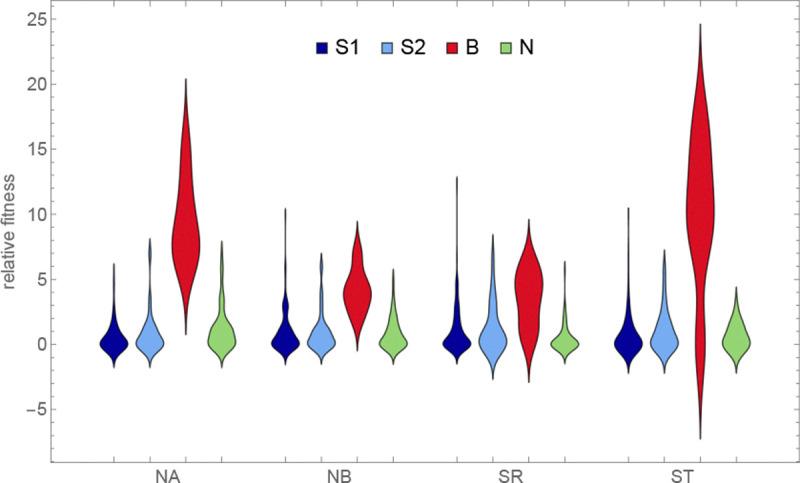
The distribution of the estimated relative fitness by genetic group (same data as in Figure 12 but rescaled by dividing by the expected frequency in the absence of selection).

The data presented in the previous section already reveal the most striking conclusion from our selection experiments, namely that the B accessions tended to outperform all other accessions in all sites (Figure 13). This unexpected result contrasts sharply with the observation that the B accessions had the lowest fecundity in all common-garden experiments (Figure 8), and obviously does not support any simple notion of local adaptation. When considering what might provide these accessions with up to a 20-fold fitness-advantage (up to 25 in ST, although this estimate may be inflated) in the selection experiments (Figure 13), we noted that some of them tend to produce very large seeds, which might be an advantage in seedling establishment, if for no other reason than that seedlings from large seeds grow faster (Clauw et al., 2022).

**Figure 14.**
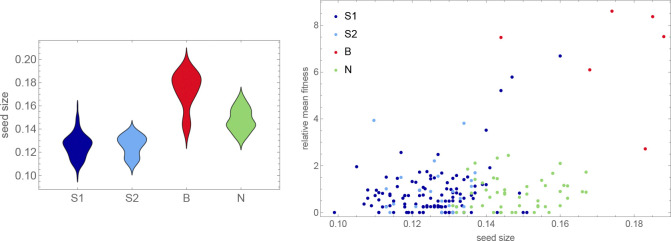
Left: The distribution of seed size (data from Clauw et al., 2022) by genetic group. Right: Mean fitness (from Figure 13) as a function of seed size.

Using existing seed size measurements, we discovered that our genetic groups did indeed differ dramatically in seed size, with the B accessions producing by far the largest seed (Figure 14). Plotting fitness against seed size confirms that there is a relationship—but it is not a simple correlation. In particular, while N accessions also have large seeds (albeit it not as large as B accessions), they have no higher fitness than S1 and S2 accessions. Large seed size seems to be necessary but not sufficient for high fitness.

**Figure 15.**
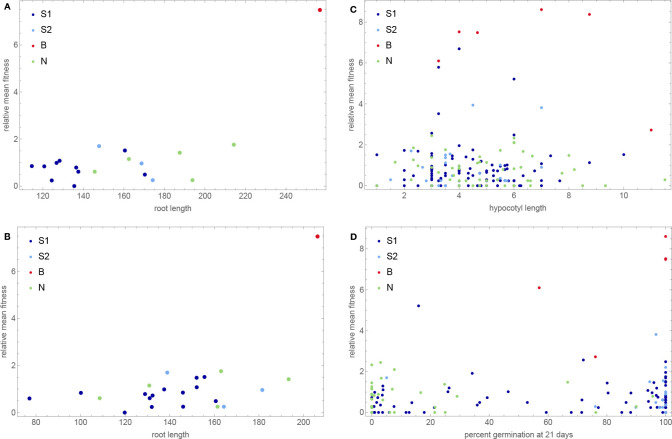
Mean fitness as functions of various phenotypes related to seedling establishment. **A.** Root length, from Slovak et al. (2020). **B.** Same as A, but corrected for seed size, which was positively correlated with early root growth. **C.** Hypocotyl elongation. **D.** Primary seed dormancy, from Kerdaffrec et al. (2016).

Inspired by these results, we examined other traits that might contribute to fitness variation, in particular those related to seedling establishment. Based on field observations, we knew that B accessions tended to have very long roots—likely a necessity for growing on a sandy beach. Wolfgang Busch’s laboratory has measured early root growth experimentally and shown that it depends on seed size, but also that it has a genetic basis that appears to be independent of seed size (Slovak et al., 2020). Their studies include a small subset of the Swedish accessions used here, and indeed we see that the one high-fitness B accession included is an outlier for rapid root growth when seed size is not accounted for, and remains at the top even when it is (Figure 15, panels A–B). This accession thus has large roots partly because it has large seeds and partly because it is genetically predisposed to grow large roots, irrespective of seed size.

Another plausible trait is hypocotyl elongation, which could be especially useful for buried seeds. We tested this experimentally, but no obvious pattern was found (Figure 15, panel C). Finally, we had already shown that B accessions tended to have high primary dormancy, whereas N accessions have very low primary dormancy (Kerdaffrec et al., 2016). We interpreted this as local adaptation to hot and dry summers on southern beaches vs adaptation to very short growing season in the north. Consistent with this, there is no obvious relationship between seed dormancy and mean fitness across our experiments (Figure 15, panel D), but the pattern seen in individual experiments does support dormancy playing a role in local adaptation (Figure 15—figure Supplement 2).

**Figure 16.**
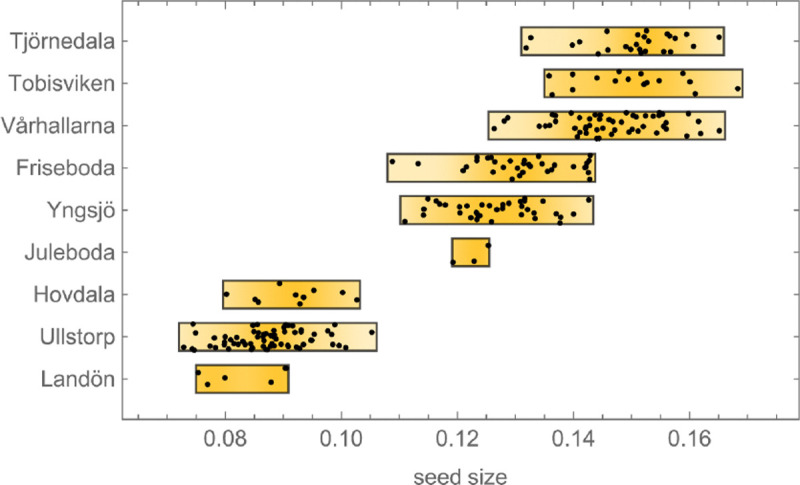
Seed size distribution for 246 additional accessions sampled from 9 sites in southern Sweden in 2017 (see Methods). The top 6 sites were located on beaches and the bottom 3 were located inland. The experimental B accessions hail from the top four sites.

Note also that, while the B accessions stand out as having very high fitness, fitness is not perfectly correlated with genetic group. In addition to five B accessions, the top right corner of the scatter plot in Figure 14 contains three S1 accessions that have lower fitness and seed size, but are still positive outliers. Two of these hail from the same beaches as the B accessions and the third had been sampled from another sandy beach—on the island of Gotland in the middle of the Baltic Sea, over 500 km away (Figure 1). To confirm that the apparent association between seed size and beach habitat is real, we measured seed size in 246 additional Swedish accessions. The pattern was confirmed (Figure 16).

#### Estimating allele-frequency changes

**Figure 17.**
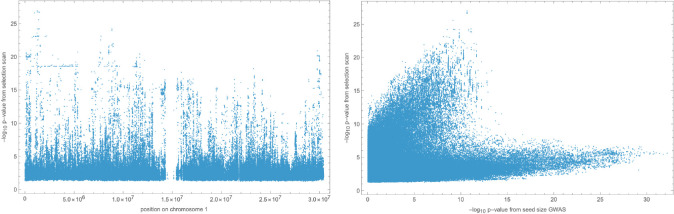
Left: results of selection scan for chromosome 1. Right: scatter plot of p-values from selection scan (all chromosomes) vs. seed size GWAS.

To gain insight into the genetic basis of fitness, we considered changes in SNP frequencies during the course of the experiment. We used basic population genetics to model changes in the absence of selection, and estimated significance using standard statistics (see Methods). Because the results across experiments were highly correlated, we combined result by simply multiplying *p*-values. The results of this selection scan can be visualized like GWAS results using a Manhattan plot, and look very similar. They are also plagued by population structure—as for GWAS results, we see horizontal bands of identical p-values reflecting extensive haplotype structure and long-range linkage disequilibrium (Figure 17). Another manifestation of population structure confounding is the curious relationship between p-values from a seed size GWAS and from the selection scan. As can be seen in the right panel of Figure 17, this distribution has two main axes. The lower one captures the total variation in seed size, which is associated with S1 and S2 vs N and B, but is not strongly associated with fitness—N accessions do not have high fitness despite their larger seeds (Figure 14). The upper one captures only the component of seed size variation that is also associated with fitness, *i.e.*, B accessions vs everyone else. Note that a large number of SNPs are highly associated with both axes, reflecting strong population structure.

However, as noted above, high fitness is not perfectly correlated with population structure, and while our SNP matrix contains 63 (out of 1,240,486) SNPs in multiple regions on all chromosomes distinguishing the B accessions from all other accessions, there are only 9 SNPs in a single 500 kb region on chromosome 1 that distinguish the B accessions plus the 3 other high-fitness beach accessions mentioned above from all other accessions. This region underlies the highest peak in the selection scan (Figure 17), and is centered on two plausible candidate genes: *YUCCA3* (AT1G04610), associated with auxin biosynthesis and early root growth (Zhao et al., 2001); and *MEE4* (Maternal Effect Embryo Arrest 4, AT1G04630), a mitochondrial protein with several embryo- and seed-related phenotypes (Pagnussat et al., 2005; Dong et al., 2016)

## Discussion

This study was designed to look for evidence of local adaptation within Sweden, and to investigate whether selection experiments would reveal important factors that had been missed by traditional common-garden experiments. It was successful in both respects. Consider:

- A subset of southern S1 accessions experienced severe overwinter mortality (and decreased fecundity among survivors) in both northern sites in one year of our common-garden experiments, presumably reflecting a harsh winter (Figure 3).
- Slugs attacked one southern site in one year of our common-garden experiments, almost exclusively feeding on S1 and S2 plants, dramatically increasing overwinter mortality and decreased fecundity among survivors (Figure 4).
- Accessions tended to have higher fecundity closer to home. Thus S1, S2, and B accessions had higher fecundity in southern than in northern sites, and the reverse was true for N accessions (Figure 9).
- This notwithstanding, S1 and S2 accessions generally had higher fecundity than N accessions across sites, and B accessions universally had low fecundity (Figure 8).
- Regardless of location, B accessions had a massive fitness advantage in all selection experiments, presumably related to seedling establishment (Figure 14).

The implied strength of selection was very large: in both the overwinter survival (Figure 3) and the selection experiment data (Figure 14), over five-fold differences in fitness between accessions were common.

We interpret these observations as reflecting at least three different evolutionary trade-offs: resistance (to stress) vs. high fecundity; seed quality (good seedling establishment) vs. seed quantity (high fecundity) and, finally; latitude vs. latitude—for unknown reasons, accessions tend to have higher fecundity closer to home. The latter sounds like textbook local adaption, but this is not the case because even though N accessions have relatively higher fecundity in the north than in the south, they still have lower fecundity than S1 and S2 accessions. Without other factors influencing fitness, they would be out-competed.

It is easy to see how a trade-off between resistance and fecundity could maintain variation, however. Our results for S1 and S2 vs. N are reminiscent of those of Oakley et al. (2023), who, through years of common-garden experiments using a RIL population generated by crossing an Italian and Swedish accession (from the same region as our N accessions), have found a similar trade-off under which Italian genotypes generally have an advantage due to higher fecundity, but suffer much higher mortality in the north in some years. In our experiments we see that while S1 and S2 accessions generally have higher fecundity, they are more sensitive to both slug and (presumably) weather-induced damage, resulting in increased mortality as well as decreased fecundity among survivors. However, to argue that this trade-off *actually* maintains variation we would need much more information about the intensity of weather- and herbivory-induced stress on the requisite spatial and temporal scales (both of which are unknown; see below)—and we would also need to know how relevant these common-garden estimates of survival and fecundity are to total fitness.

The answer to the latter question is far from obvious, as the results from our selection experiments make abundantly clear. Totally unexpectedly, we found that B accessions from the very south of Sweden had a fitness advantage in all sites, which is especially notable given their consistently low fecundity in our common-garden experiments. The implied selection strengths are enormous, with accessions increasing their frequency up to 20-fold over little more than a single life-cycle. While we do not know what caused these fitness differences, it is reasonable to hypothesize that it involves components not assayed in the common-garden experiments, in particular seedling establishment and competition. This hypothesis is strengthened by the observation that fitness appears to be correlated with a number of traits related to seedling establishment, like seed size (Figure 14), dormancy and root growth (Figure 15).

A trade-off between seed size and seed number is not surprising, but what could maintain variation for such traits? We would suggest that classical r/K selection plays a role (as has also been suggested by Bastias et al., 2024; Lo et al., 2024). The S1 and S2 accessions were mostly collected in small, ephemeral patches directly or indirectly created by human activity. The N accessions were mostly collected in patches created by ongoing natural erosion on south-facing slopes, many of which had harbored *A. thaliana* for at least a century according to local floras. The B accessions, finally, were collected in very large patches on beaches—very stable environments dating back to the last glaciation. It is likely that the existence of S1 and S2 accessions is far more uncertain, and that they therefore have become adapted to a more weedy, r-selected life history characterized by smaller seed—similar to what has been argued for the global species distribution (Bastias et al., 2024; Lo et al., 2024). Meanwhile B accessions might out-compete other accessions in any given patch, but will go extinct with that patch because they are poor at dispersal. The observed distribution of seed size supports this notion, with S1 and S2 accessions having significantly smaller seeds than B and N accessions (Figure 14), although the very large seed size of the B accessions probably also reflects more direct adaptation to the extreme beach environment. Whatever the mechanism, the result is a dramatic competitive advantage in our selection experiments.

To summarize, while we uncovered plenty of evidence of fitness-related variation consistent with local adaptation, we were also reminded of how complex the adaptive landscape is, and how difficult it is to measure fitness. Even our selection experiments, which included the full life cycle, measured total fitness conditional on finding yourself in a plot where you are able to compete. If the kind of r-selection discussed above is important, then dispersal ability and seed banks would also play a major role (see, *e.g.*, Fakheran et al., 2010). Designing experiments that include these fitness components would be challenging even if we knew the relevant spatial and temporal scales, which we assuredly do not (for other examples, see de la Mata et al., 2024; Schmitz et al., 2024).

Our study also illustrates the challenges facing efforts to understand evolutionary change from the molecular to the ecological level. For example, there is currently much discussion about the “genetic architecture of adaptation", but how will we quantify that if we cannot measure fitness? We are on more solid ground if we restrict ourselves to fitness-related traits—which can surely be measured, as we have shown in this study—but even here the task is formidable, because genetically dissecting a trait is not easy. Identifying causal polymorphisms requires years of experimental work that is only feasible in a few model organisms, and even estimating marginal effects of individual loci in the presence of population structure and selection is very hard. Genome-wide linkage disequilibrium is a direct consequence of population structure (as first realized by Wahlund, 1928), and leads to biased effect-size estimates across the genome due to correlations with causative polymorphism. The problem is worse for phenotypes that are locally adaptive because the causal polymorphisms will by definition be correlated with population structure—leading to stronger genome-wide confounding, as well as linkage disequilibrium between causative loci (reflecting selection as well as population structure, see Ohta, 1982). Established statistical methods for addressing “population structure" exist but do not deal well with this problem (reviewed in Vilhjálmsson and Nordborg, 2012). As a consequence, while we are able to understand the architecture of very simple traits (like anthocyanin production, see Figure 10), more complex traits remain elusive. Even in a model organism like *A. thaliana*, where tissue-specific expression data and mutant phenotypes exist for most genes, guessing which gene might be responsible for an association is generally not fruitful, and estimating effect sizes requires experiments. An alternative to GWAS is various forms of scans for selection, but it is not clear how one quantifies "genetic architecture" based on such data—and if deducing molecular mechanism is difficult for GWAS, it is far worse when we do not even begin with a phenotype.

Finally, our study points to a critical area of research for population genetics, namely understanding the scale of adaptation. Models are meant to help us make sense of the world, and over a century of theoretical population genetics has provided us with a rich modeling framework to this end. All models rely on simplifying assumptions, but this is by design—a feature rather than a bug—as they can still be useful as long as we understand their limitations. To take a simple example, Hardy-Weinberg equilibrium relies on a plethora of assumptions, but is useful precisely because we know why it usually fails—and on what scale.

When it comes to adaptation, things are more complex. Consider resistance to a new pathogen. In the simplest case this will lead to a classic “selective sweep" affecting a genomic region the size of which is determined by the ratio of the speed of the sweep (determined by the strength of selection) and the recombination rate (Maynard Smith and Haigh, 1974). This basic intuition holds if we make the model more complicated: selfing will decrease the effective recombination rate (Nordborg, 2000), for example, and population structure will impede selection, making it more likely that resistance emerges independently (Ralph and Coop, 2015). In a scenario full of strong local adaptation, as appears to be the case for *A. thaliana* in Sweden, a resistance allele would be further impeded by selection against migrants, and might have to run a gauntlet of linkages to genes directly involved in local adaptation—a scenario similar to gene flow in a hybrid zone (Barton and Bengtsson, 1986; Barton and Navarro, 2002; Nordborg and Innan, 2003). We have the framework for modeling this, but too little knowledge about migration and selection to produce useful predictions. As a result, we do not know how long it would take a resistance allele to sweep through *A. thaliana*—if indeed this would be possible on a meaningful time scale. Rectifying this is not easy, but massive numbers of genomes densely sampled within and between species should at least help shed light on migration, past and present (Osmond and Coop, 2024). At a time when the global temperature appears to be rising almost a quarter of degree per decade, this information is likely to be useful.

## Methods

### Genotypes

All 200 accessions were re-sequenced to confirm identity. This lead to the discovery of minor discrepancies most likely reflecting residual heterozygosity in the the original lines, and we generated a new SNP matrix partly to reflect this (as well as improvements in SNP-calling algorithms, see Brachi et al., 2022).

In addition, during the course of writing this paper, we discovered that two of our accessions are not what they are supposed to be. These two accessions (1435 and 6180) are annotated as originating from northern Sweden, but several lines of evidence demonstrate that this is false:

1. their SNP genotypes do not match early genotypes for these accessions Horton et al. (2012), nor genotypes in old crosses made with these accessions;
2. in our analysis population structure, they bear no resemblance to other N accessions and instead cluster with S1 accessions;
3. in particular, the pattern of haplotype sharing indicates that they are very closely related (possibly part of an inbred sibship) with accession 5829, supposedly collected at Ales Stenar in the extreme south of Sweden.

Since all of our analyses are based on the actual genotypes, our results are unaffected by this.

### Population structure

To explore patterns of population structure in our sample of 200 accessions from Sweden, we generated a set of 124 071 LD-pruned SNPs with minor allele frequencies (MAF) of at least 3%. Minor allele frequencies filtering and LD pruning were performed in plink v1.9 (Chang et al., 2015) using the options -maf set to 0.03, and –indep-pairphase with a window of 5kb, a step size of 10 SNPs and a $r^{2}$ threshold of 0.2.

Using this set of SNPs we computed a standard genetic distance among accessions in R using the read_plink function from the package genio v1.1.2 to read-in the data, and the dist.gene function from the package ape v5.7–1 (Paradis and Schliep, 2019). The neighbor-joining tree was created with the nj function.

We performed admixture analyses with the same set of SNPs using the R package LEA 3.12.2 (Frichot and Francois, 2015). Specifically we used the snmf function and performed 10 runs for K ranging from 1 to 8. Ploidy was set to 2, alpha to 100. For each K we picked the run with the minimum cross entropy.

**Figure 18.**
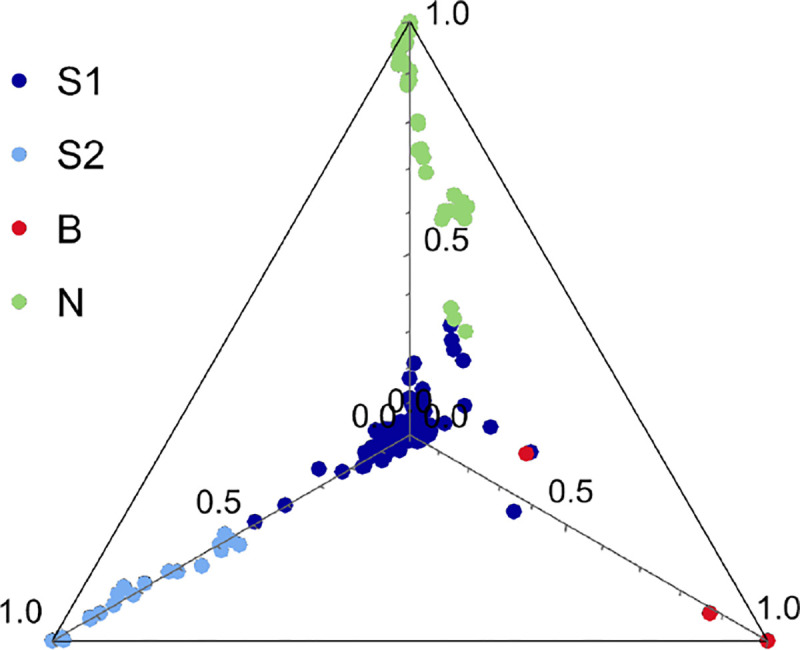
The 200 accessions plotted in the simplex space generated by the K=4 admixture proportions.

To present the results from our study we picked K=4 as it the largest value for which “pure" accessions are found (Figure 1—figure Supplement 1). Accessions were assigned to one of four group based on their largest components. As shown in Figure 18, many accession are intermediate between S1 and the other three groups, and there is also some admixture between S1, B, and N.

### Common-garden experiments

#### Experimental design

These experiments are explained in detail in Brachi et al. (2022). Briefly, seed for 200 accessions of *A. thaliana* from Sweden were produced together in a randomized design in the greenhouse of the University of Chicago under long-day conditions with a 12-week vernalization period at 4°C to induce flowering. These seeds were then used to set up common-garden experiments, repeated over two seasons in four locations in Sweden:

**NA** South-facing slope near Ådal (lat. 62.862, lon. 18.331)

**NM** Agricultural field near Ramsta (lat. 62.85, lon. 18.193)

**SR** Agricultural field near Rathckegården (lat. 55.906, lon. 14.260)

**SU** Agricultural field near Ullstorp (lat. 56.067, lon. 13.945)

Each experiment was organized in three complete blocks, each including eight replicates of each accession. Seeds were sown in trays of 66 pots, each measuring 4 cm in diameter, in a mix of 90% standard greenhouse soil and 10% local soil. Seedlings were allowed to establish outside under shelter and were thinned to single pot before being moved to the field sites and laid on tilled soil. Plants were watered once upon installation. The same dates were used in both years (Table 1).

### Phenotyping

We scored the presence and absence of each plant in fall and in spring, just after snow melt, and used these data to compute overwinter survival as a simple binary variable. Note that in the fall of 2012 we collected from 0–2 replicates per block to study gene expression and methylation (data not used in this study), and in the spring of 2012 and 2013, we also sacrificed two replicates (per accession and block) for DNA extraction and microbial community analyses (Brachi et al., 2022). The data included with this paper reflect this.

In one experiment (SR 2011–12), the plants suffered damage from slugs, which we scored in November 2011, using *ad hoc* scale: 0 (no plant), 1 (undamaged plant), 2 (mild damage) and 3 (extensive damage).

In addition, we photographed trays in both fall and spring. The images taken in the fall were used to investigate rosette sizes and purpleness using high-throughput image analyses (see the Rosette purpleness section, below).

The remaining replicates for each accessions were left to complete their life cycle in the field. With very few exceptions, all plants flowered within a two-week period in spring in each experiment (flowering is several weeks later in the north), and our records were insufficiently fine-grained to consider genetic difference.

When seeds were mature in late spring, all plants remaining in the experiments were harvested for fecundity estimation. This was achieved using high-throughput image analysis, validated by hand measurements (details in Brachi et al., 2022).

### Analysis

#### Survival

The overwinter survival data were analyzed in a generalized mixed model framework using the lme4 R package v 1.1–35.5 (Bates et al., 2015). Specifically we used the glmer function with optimizer set to bobyqa and the iterations to 2 × 10^5^ with option maxfun to fit models of increasing complexity. The simplest model included a single random block effect to explain survival, and the most complex model is described by:

(1) $${ows}_{i}\;∼Binomial(n=1,{prob}_{\text{ows}=1}={\hat{P}}_{i})\;log\left[\frac{{\hat{P}}_{i}}{1\;-\;{\hat{P}}_{i}}\right]\;=\alpha +\beta_{\text{site}}^{⊤}X_{\text{site},i}+\beta_{\text{year}}^{⊤}X_{\text{year},i}+\beta_{\text{site:year}}^{⊤}X_{\text{site:year,}i}+\alpha_{j[i]}+\gamma_{k[i]}\;\alpha_{j}\;∼N\left(0,\sigma_{\alpha }^{2}\right),\;\text{for}\;\text{exp:year:block}\;j=1,\;\ldots \;,J\;\gamma_{k}\;∼N\left(0,\sigma_{\gamma }^{2}\right),\;\text{for}\;\text{exp:year:acc}\;k=1,\;\ldots \;,K$$

In this model, ows describes the overwinter survival of the $i^{\text{th}}$ plant: 0 for dead plants, 1 for plants still alive. ${\hat{P}}_{i}$ is the probability of surviving winter and is modeled using a logit link function. α is the model intercept and β s are vectors of fixed effect coefficients for sites, years, and their interaction. Block effects within sites and years are captured by a random intercept $\alpha_{j}$, which is assumed to follow a normal distribution. Accession effects within sites and years are captured by a second random intercept term $\gamma_{k}$, also assumed to follow a normal distribution. This model fitted the data better (based on AIC, BIC and log-Likelihood) than a model without the random accession effect within site and year $\left(\gamma_{k}\right)$. Accessions effects or BLUPS (Best Linear Unbiased Predictions, $\gamma_{k}$) were computed from this model and used in GWAS.

To compare survival in the two northern sites in 2011–22 (Figure 3), we used a simple liability-threshold model, as also used by the logit-transformation above. Briefly, we assume liability is Gaussian with accession i having a genetically determined $N\left(\mu_{i},\sigma_{NA}\right)$ or $N\left(\mu_{i},\sigma_{NM}\right)$ and that the probability of survival is the cumulative probability up $\tau_{NA}>\tau_{NM}$ (reflecting the harsher environment in NM). Using this model, the relationship between the survival probabilities in NM and NA can be written

(2) $$p_{NM,i}=\frac{1}{2}erfc\left(\frac{\alpha }{\sqrt{2}}+\beta \;{erfc}^{-1}\left(2p_{NA,i}\right)\right),$$

where $\alpha =\left(\tau_{NA}-\tau_{NM}\right)/\sigma_{NM},\beta =\sigma_{NA}/\sigma_{NM}$, and erfc is the complementary error function. We fit this equation to the data using NonlinearModelFit in Mathematica.

#### Herbivory damage

Herbivory damage (due to slugs and scored in SR 2011–12 only) was modeled as the number of heavily damaged plants (score 3 against the rest) per accession and block, and the number of plants per accession and block with no damage (score 1 against the rest) using equation 3.

(3) $$H_{i}\;∼Binomial(n={total}_{i},{prob}_{s1=1}={\hat{P}}_{i})\;log\left[\frac{{\hat{P}}_{i}}{1\;-\;{\hat{P}}_{i}}\right]\;=\alpha +\beta_{\text{block}\;}^{⊤}X_{\text{block}\;,i}+\gamma_{k[i]}\;\gamma_{k}\;∼N\left(0,\sigma_{\gamma }^{2}\right),\;\text{for}\;\text{acc}\;j=1,\;\ldots \;,\;J$$

In the model, $H_{i}$ refers to the number of heavily damaged (or undamaged plants depending on the model) among plants in the $i^{\text{th}\;}$ set of plants representing one accession in one block, and ${total}_{i}$ is the number of observed plants in this set. ${\hat{P}}_{i}$ is the predicted probability of damage (or of being undamaged) and is modeled using a logit link function. α is the intercept. $\beta_{\text{block}}$ is the vector of fixed-effect coefficients for the block, and $X_{\text{block,}i}$ defines the block for the $i^{th}$ set of plants. The term $\gamma_{k}$ represents the random intercepts capturing accession effects and follows a normal distribution. Accession effects (BLUPs, $\alpha_{j}$) were extracted from the models for heavily damaged plants and undamaged plants.

#### Rosette purpleness

Trays in the common-garden experiments were photographed as follows:

- SR on the 09/29/2011, 11/20/2012 and 11/25/2012
- SU on the 11/01/2011, 09/11/2011 and 11/22/2012
- NA and NM on 09/29/2012

Tray images were cropped by hand to the edge of the trays, and plants were then segmented automatically using custom scripts based on the EBImage R package (Pau et al., 2010). Briefly, the image segmentation starts by normalizing the images. Pixels are then clustered with the R function clara from the package cluster, with K=3. The cluster with the highest average green value is considered to represent mostly plants. Pixels from the other clusters were masked and the segmentation of plant was refined by successive erosion, expansion and blurring. The result consists of tray images with up to 66 plants on a white background. Images were all manually checked and the segmentation corrected when needed in Photoshop CS6 (*i.e.*, separating touching plants or removing remains of the background). These images were then used to measure the color composition of individual rosettes (and their size). First, white and black pixels were removed from the segmented images as they carry no information. Colors from the whole image were then reduced to 16 color groups, using k-means clustering (based on the RGB values). The frequency spectrum of 16 colors was then computed for each plant on the image and each plant was assigned to a position on the tray in terms of rows (1–6) and columns (1–11). The resulting table consists, for each tray, of the plant coordinates on the tray (used to match with accession identity) and 16 columns of pixel counts corresponding to the 16 colors. Simultaneously, the RGB values of the 16 colors identified on each tray were recorded in another table.

The RGB values of all observed colors across all trays in all experiments (16 colors per tray, amounting to 17,152 unique colors) were then summarized using a principal components analysis of the normalized R, G and B values. In this analysis the purple to green gradient is well captured by the first component, which explains 75% of the variance. The coordinates of the 16 colors from each tray along this component are then multiplied by the proportions of pixel of these colors found in each plant. The resulting vector quantifies the purpleness in 32,154 rosette images.

To investigate the effects of genetic variation on plant purpleness, we modeled purpleness using the R package lme4, using the following model:

(4) $$\begin{array}{l} {rcolor}_{i}∼N\left(\mu ,\sigma^{2}\right)\\ \;\mu =\alpha_{j[i],l[i]}+\gamma_{k[i]}+\beta_{\text{site}\;}^{⊤}X_{\text{site},i}+\beta_{\text{year}\;}^{⊤}X_{\text{year},i}+\beta_{\text{site:year}\;}^{⊤}X_{\text{site:year,}i}\\ \;\gamma_{k}∼N\left(\mu_{\gamma_{k}},\sigma_{\gamma_{k}}^{2}\right),\;\text{for}\;\text{site:year:acc}\;k=1,\;\ldots \;,\;K\\ \;\alpha_{j}∼N\left(\mu_{\alpha_{j}},\sigma_{\alpha_{j}}^{2}\right),\;\text{for}\;\text{site:year:block}\;\;j=1,\ldots \;,\;J\\ \;\alpha_{l}∼N\left(\mu_{\alpha_{l}},\sigma_{\alpha_{l}}^{2}\right),\;\text{for}\;\text{site:year:date}\;I=1,\;\ldots \;,\;L \end{array}$$

In this model, ${rcolor}_{i}$ is the purpleness of the $i^{\text{th}}$ plant. The terms of the model are largely identical to the models used above. Purpleness variation is explained by fixed effects for site and year effects and their interactions (β), as well as random intercept effects for accessions ($\gamma_{k}$) and blocks within sites and years $\left(\alpha_{j}\right)$. We added a random intercept terms for photography date $\left(\alpha_{l}\right)$ as some experiments were photographed twice the same year. This model was used to generate BLUPs used in GWAS and to investigate genetic correlations with other traits.

#### Fecundity

The per-plant fecundity estimates were derived from image analysis of mature stems. Note that the raw areas occupied by mature plants on images (our fecundity estimate) was log-transformed (log_10_) prior to analyses. All plants for which we retrieved no stem, or only fragments were removed from this analysis (there are no zeros in the data).

Estimates were again modeled using a linear mixed model. We proceeded exactly as we did for the overwinter survival, starting from a simple model including only a random block effect, to incrementally complex models. Again, the most complex model provided a better fit (based on AIC, BIC and log-likelihood):

(5) $$\begin{array}{l} {log}_{10}(\;\text{fecundity}\;{)}_{i}∼N\left(\mu ,\sigma^{2}\right)\\ \;\mu =\alpha_{j[i]}+\gamma_{k[i]}+\beta_{\text{site}}^{⊤}X_{\text{site},i}+\beta_{\text{year}}^{⊤}X_{\text{year,}i}+\beta_{\text{site:year}}^{⊤}X_{\text{site:year,}i}\\ \;\alpha_{j}∼N\left(\mu_{\alpha_{j}},\sigma_{\alpha_{j}}^{2}\right),\;\text{for}\;\text{site:year:block}\;j=1,\ldots ,\;J\\ \;\gamma_{k}∼N\left(\mu_{\alpha_{k}},\sigma_{\alpha_{k}}^{2}\right),\;\text{for}\;\text{site:year:acc}\;k=1,\ldots ,\;K \end{array}$$

Terms in this model are described for other models. Model residuals were checked for heteroskedasticity and large deviation from normality.

As for overwinter survival, BLUPs were extracted for accessions within sites and years and used as phenotypes in GWAS.

### GWAS

Accession effects (BLUPs) obtained from generalized linear models described above were used in GWAS. The SNP matrix used was filtered to include only bi-allelic SNPs with MAF ≥ 3%. Traits were mapped for sites and years separately using the generalized linear models (GLM, no control for population structure) and mixed-models (MLM, controlling for population structure using a kinship matrix) implemented in rMVP v1.0.6 (Yin et al., 2021). Custom plotting functions based on ggplot2 were used for generating Manhattan plots, including zoom-ins.

### Experimental evolution experiments

#### Installation

Our experimental evolution experiments were carried out at four sites, two in the north, and two in the south, with one site in each region associated with a common garden experiment (Figure 1):

**NA** South-facing slope near Ådal (lat. 62.862, lon. 18.338)

**NB** South-facing slope near Barsta (lat. 62.87, lon. 18.381)

**SR** Agricultural field near Rathckegården (lat. 55.906, lon. 14.260)

**ST** Sandy beach/pasture near Tjörnedala (lat. 55.60, lon. 14.304)

The sites were chosen to be realistic *A. thaliana* habitats, but without *A. thaliana* visibly present. Within each site we delimited four to five replicate 1 m^2^ plots marked with large metal nails hammered into the ground until invisible. Thus the plots themselves were invisible, but could be relocated using a metal detector. Each plot consisted of 3 to 4 contiguous 60 × 40 cm subplots arranged to match the terrain. Using seeds from the same lots used for the common-garden experiments (see above), we prepared tubes containing 40 seeds for each of the 200 accessions using an Elmor C3 seed counter. Prior to dispersal, seed from each tube were mixed with sterile sand of about the same granularity as Arabidopsis seeds, and transferred to ≈ 20 ml plastic vials with holes drilled in the caps. This allowed for homogeneous dispersal of 40 × 200 = 8,000 seeds in each subplot, guided by a 60 × 40 cm frame with a string mesh delimitating 10 × 10 cm squares. The dispersal was repeated 3 times over three weeks in 2011 to maximize the chances of successful establishment. In the north (NA and NB), we dispersed seeds August 4th–6th, 15th–16th and 23rd–24th. In the south (SR and ST), we dispersed seeds August 28–30, September 7–8 and September 15–16. In total we thus dispersed 120 seeds per accession per m^2^.

#### Genotyping

Individual plants we sampled randomly using a grid in spring 2013. At least 70 plants were sampled per (surviving) plot in each patch. Multiplexed DNA libraries were prepared using the Illumina Nextera^™^ Kit. The standard Nextera library construction protocoal was changed to handle reduced volumes. Tagmentation reaction was set up to 2.5 μl final volume with 2.5 ng of input DNA. For PCR amplification and multiplexing we used Illumina Nextera Dual primers. Size selection and PCR clean-up were performed with Agencourt AMPure XP Beads (Beckman Coulter). After PCR enrichment, libraries were validated with Fragment Analyzer^™^ Automated CE System (Advanced Analytical) and pooled in equimolar concentration for 96X-multiplex. Libraries were sequenced on Illumina HiSeq^™^ V4 Analyzers using manufacturer’s standard cluster generation and sequencing protocols in 125 bp PE mode at the Vienna Biocenter Core Facilities using standard Illumina paired-end protocols. We produced on the order of 1–2 million reads for each sample with an average read-length of 100 bp. These reads were mapped to the reference TAIR10 genome using bwa-mem with default parameters. We genotyped 2.3 million previously identified SNP using bcftools with default parameters (base quality threshold of 30 and read mapping quality of 10). The nextflow pipeline for SNP calling is available at https://github.com/Gregor-Mendel-Institute/nf-haplocaller.

**Figure 19.**
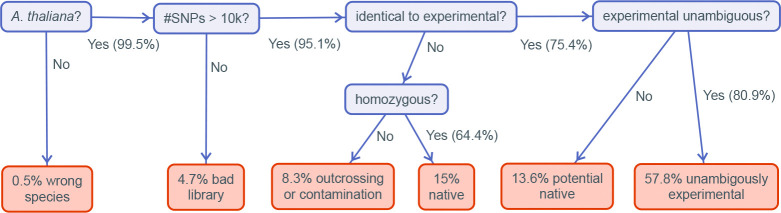
Workflow for classifying selection experiment samples.

In order to assign each sample to one of the 200 experimental accession, the workflow summarized in Figure 19 was used. We outline each step below.

1. Many samples were very small (collection was done using tweezers), and there was a clear risk of sampling the wrong species. To quickly eliminate such samples, we mapped reads to *A. thaliana* centromeric repeats and organellar genomes.
2. We estimated the minimum number of SNPs required for genotyping the samples using the SNPmatch “simulation” function (Pisupati et al., 2017). With the exception of two pairs of accessions, 5k SNPs random were sufficient to distinguish all accession. To be conservative, we set a threshold of 10k SNPs.
3. We used SNPmatch and an empirically derived mismatch threshold of 0.015 (see Pisupati et al., 2017) to assign samples to experimental lines.
4. Of the roughly 1/4 of samples that did not match an experimental accession, roughly 1/3 showed extensive heterozygosity that could either reflect recent outcrossing or sample contamination (the quality of our data was not sufficient to investigate this further, nor were we able to identify parents of putatively outcrossed individuals). The remaining 2/3 were inbred accessions that we classified as “natives".
5. Of the 3/4 of samples that did match an experimental accession, 19% matched an accession that might also have been present as native in the experimental site where the sample was taken (a “potential native"; see next section) while the remaining 81% were unambiguously experimental samples.

#### Potential natives

The observation that all four experimental sites contained obvious natives alerted us to the possibility that our results could be biased by the presence of cryptic natives, *i.e.*, native members of one of our experimental accessions. This would be far more likely to happen for an experimental accession that was originally collected close to the experimental site, because, with the notable exception of North America, where the species is recently introduced, *A. thaliana* is characterized by strong isolation-by-distance and the probability of finding identical individuals decays rapidly with distance (***Platt et al., 2010a***).

**Figure 20.**
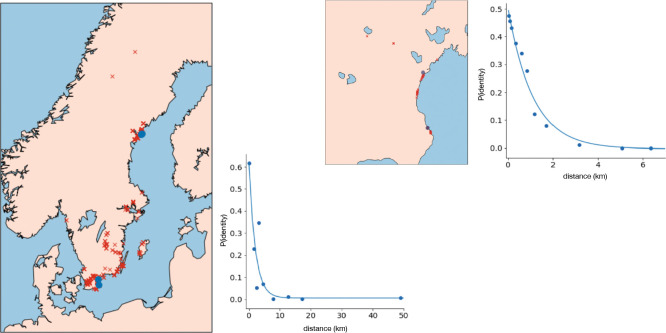
Isolation-by-distance in Swedish *A. thaliana*. On the left, sample locations and the decay in pairwise identity as a function of distance using the data of ***Platt et al.*** (***2010a***); on the right, the same plots for the new samples from Skåne (Figure 16). The blue dots show the sites used in the evolution experiments.

This pattern can be seen in Figure 20, where we plot isolation-by-distance for the old data by ***Platt et al.*** (***2010a***), and also for the new “beach"-centered collection used to examine the distribution of seed size (Figure 16). The new data confirm the conclusion of ***Platt et al.*** (***2010a***), but has higher resolution due to much denser sampling. Identical genotypes are very rarely seen more than a few km apart, although exceptions exist in the south (where they are likely due to human dispersal). In the almost continuous beach population we sampled, the probability effectively declines to zero ($p=3.9\times 10^{-6}$) at 5.6 km.

Taking 5.6 km as a cut-off, we see that results for a total of 11 accessions could have been be confounding by cryptic natives in the northern sites, and results for 8 accessions could have be confounding at the ST site. No experimental accession was sampled close enough to the SR site for it to be plausible that result were confounded by cryptic natives.

As shown in Figure 12, we find convincing evidence that two data points (the Bar1 accession in NB, and the Vår2–6 accession in ST) were indeed confounded. However, more importantly, the main result of these experiments, namely that the same beach accessions outperformed inland accessions in all sites, could not possibly be explained by this kind of confounding.

#### Modeling allele-frequency change

The experimental sites were sown in fall 2011, and seedlings were allowed to establish normally and set seed in spring/summer 2012, giving rise to a second generation that germinated in fall 2012. Survivors were then sampled in spring 2013. While we cannot rule out that some samples might be dormant survivors of the original sowing rather than their descendants, we note that there was a substantial flowering population in 2012 in all sites.

Either way, from the perspective of understanding how allele-frequencies would change in the absence of selection, the population of (experimental) individuals in spring 2013 can be modeled as Wright-Fisher multinomial sample of size $N_{e}$ from a starting population where all 200 accessions had equal frequency. At the level of SNPs, the population frequency of an allele with starting frequency $p_{0}$ will thus be binomially distributed with parameters $N_{e}$ and $p_{0}$. This frequency is what we then estimate using *bona fide* binomial sampling with (known) sample size n. Let Δp be the difference between our estimate and the original allele frequency $p_{0}$. In the absence of selection, using basic probability theory, we have E[Δx]=0 and

$$Var[\Delta x]=\left(\frac{1}{n}+\frac{1}{N_{e}}+\frac{1}{nN_{e}}\right)p_{0}\left(1-p_{0}\right)\approx \left(\frac{1}{n}+\frac{1}{N_{e}}\right)p_{0}\left(1-p_{0}\right),$$

**Figure 21.**
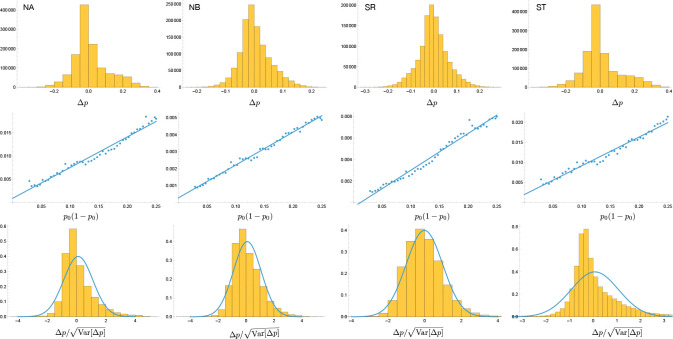
Top: the distribution of Δp in the four selection experiments. Middle: the variance of Δp as a function of $p_{0}\left(1-p_{0}\right)$. Bottom: the distribution of Δp scaled by its standard deviation. The curves are PDFs of normal distributions with the observed mean and variance 1.

*i.e.*, allele frequencies should only change due to random sampling (in this case “drift" plus literal random sampling). As shown in Figure 21, mean Δp is indeed close to zero, and the variance is a linear function of $p_{0}\left(1-p_{0}\right)$ as predicted by the equation above. Since we know the sample size n—it was 171, 199, 208 and 137 for NA, NB, SR, and ST, respectively—we can estimate the corresponding $N_{e}$ to be 17, 70, 34, and 16 from the fitted slope in the figure.

To identify Δp values too large to be due to drift, we divide them by the estimated standard deviation for the corresponding $p_{0}$, and calculate the significance of deviations from zero using a standard normal distribution. Note that the mean is not zero, reflecting the fact that allele-frequencies are biased towards a German reference genome. More importantly, there are clear genome-wide deviations reflecting strong selection and population structure (as is also evident in Figure 17).

## Supplementary Material

Supplement 1 **Figure 1—figure supplement 1.** Population structure in the 200 Swedish accessions.
**Figure 1—figure supplement 2.** The correlation of allele frequencies between the genetic groups.
**Figure 1—figure supplement 3.** Photos of field sites: common gardens
**Figure 1—figure supplement 4.** Photos of field sites: selection experiments
**Figure 2—figure supplement 1.** The distribution of survival probabilities across experiments by group.
**Figure 4—figure supplement 1.** Overwinter survival was also affected by underlying susceptibility to stress.
**Figure 5—figure supplement 1.** Manhattan plots without structure correction.
**Figure 5—figure supplement 2.** Manhattan plots with structure correction.
**Figure 5—figure supplement 3.** Zoom-in on *AOP* region.
**Figure 5—figure supplement 4.** Zoom-in on *SVP* region.
**Figure 7—figure supplement 1.** The distribution of each PC by group.
**Figure 7—figure supplement 2.** Positions of accessions in PC1-PC2 space.
**Figure 7—figure supplement 3.** Positions of accessions in PC2-PC3 space.
**Figure 8—figure supplement 1.** Accession fecundity is correlated with overwinter survival.
**Figure 11—figure supplement 1.** Manhattan plots for fecundity GWAS without correction for structure.
**Figure 11—figure supplement 2.** Manhattan plots for fecundity GWAS with correction for structure.
**Figure 12—figure supplement 1.** Same as Figure 12, but with the two obvious outliers removed to show remaining data better.
**Figure 15—figure supplement 1.** Fitness as a function of hypocotyl elongation in each experiment.
**Figure 15—figure supplement 2.** Fitness as a function of primary seed dormancy in each experiment.
**Figure 17—figure supplement 1.** Zoom-in of selection scan for chromosome 1 peak.
**Figure 17—figure supplement 2.** Result of selection scan for chromosomes 2–4.
**Figure 19—figure supplement 1.** The number of samples in each category per site and plot.

## Figures and Tables

**Table 1.** Sowing and installation date for common-garden experiments. Blocks A-C were sown with two-day intervals.

	2011		2012	
	sowing (blocks A-C)	field installation	sowing (blocks A-C)	field installation

NA	8/8–8/12	8/25	8/8–8/12	8/25
NM	8/7–8/11	8/24	8/7–8/11	8/24
SR	9/1–9/5	9/18	9/1–9/5	9/18
SU	8/31–9/4	9/17	8/31–9/4	9/17
