# Supplementary material for: Life-history trade-offs explain local adaptation in Arabidopsis thaliana
Source: bioRxiv. 2025 May 23:2025.05.18.654693. Preprint. [Version 1] doi: 10.1101/2025.05.18.654693 (PMC12139787; doi:10.1101/2025.05.18.654693)
Supplement: Supplement 1 — Figure 1—figure supplement 1. Population structure in the 200 Swedish accessions. Figure 1—figure supplement 2. The correlation of allele frequencies between the genetic groups. Figure 1—figure supplement 3. Photos of field sites: common gardens Figure 1—figure supplement 4. Photos of field sites: selection experiments Figure 2—figure supplement 1. The distribution of survival probabilities across experiments by group. Figure 4—figure supplement 1. Overwinter survival was also affected by underlying susceptibility to stress. Figure 5—figure supplement 1. Manhattan plots without structure correction. Figure 5—figure supplement 2. Manhattan plots with structure correction. Figure 5—figure supplement 3. Zoom-in on AOP region. Figure 5—figure supplement 4. Zoom-in on SVP region. Figure 7—figure supplement 1. The distribution of each PC by group. Figure 7—figure supplement 2. Positions of accessions in PC1-PC2 space. Figure 7—figure supplement 3. Positions of accessions in PC2-PC3 space. Figure 8—figure supplement 1. Accession fecundity is correlated with overwinter survival. Figure 11—figure supplement 1. Manhattan plots for fecundity GWAS without correction for structure. Figure 11—figure supplement 2. Manhattan plots for fecundity GWAS with correction for structure. Figure 12—figure supplement 1. Same as Figure 12, but with the two obvious outliers removed to show remaining data better. Figure 15—figure supplement 1. Fitness as a function of hypocotyl elongation in each experiment. Figure 15—figure supplement 2. Fitness as a function of primary seed dormancy in each experiment. Figure 17—figure supplement 1. Zoom-in of selection scan for chromosome 1 peak. Figure 17—figure supplement 2. Result of selection scan for chromosomes 2–4. Figure 19—figure supplement 1. The number of samples in each category per site and plot. [file media-1.pdf]

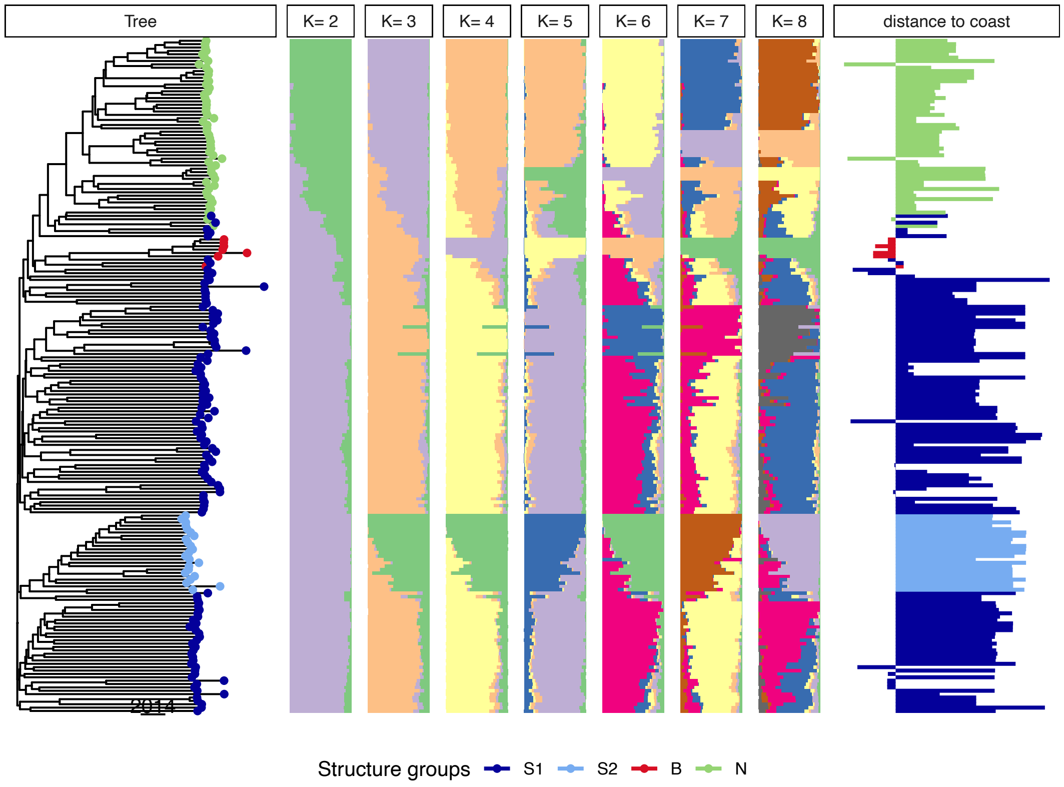

Figure 1—figure supplement 1. Population structure in the 200 Swedish accessions. On the left is a Neighbor-Joining tree of all accessions included in this study. Tree tips are colored according to the structure groups used in this paper. The genetic distance between accessions was computed using 124,071 LD-pruned SNPs. The following panels show ADMIXTURE results for different values of K. Note that for $K > 4$, only “admixed" colors are added. The right-most panel shows the distance to the Baltic Sea for each accession, in hundreds of meters and presented on a $\log_{10}$ scale. Hence, leftward pointing bars highlight accessions collected less than 100 m from the coastline.

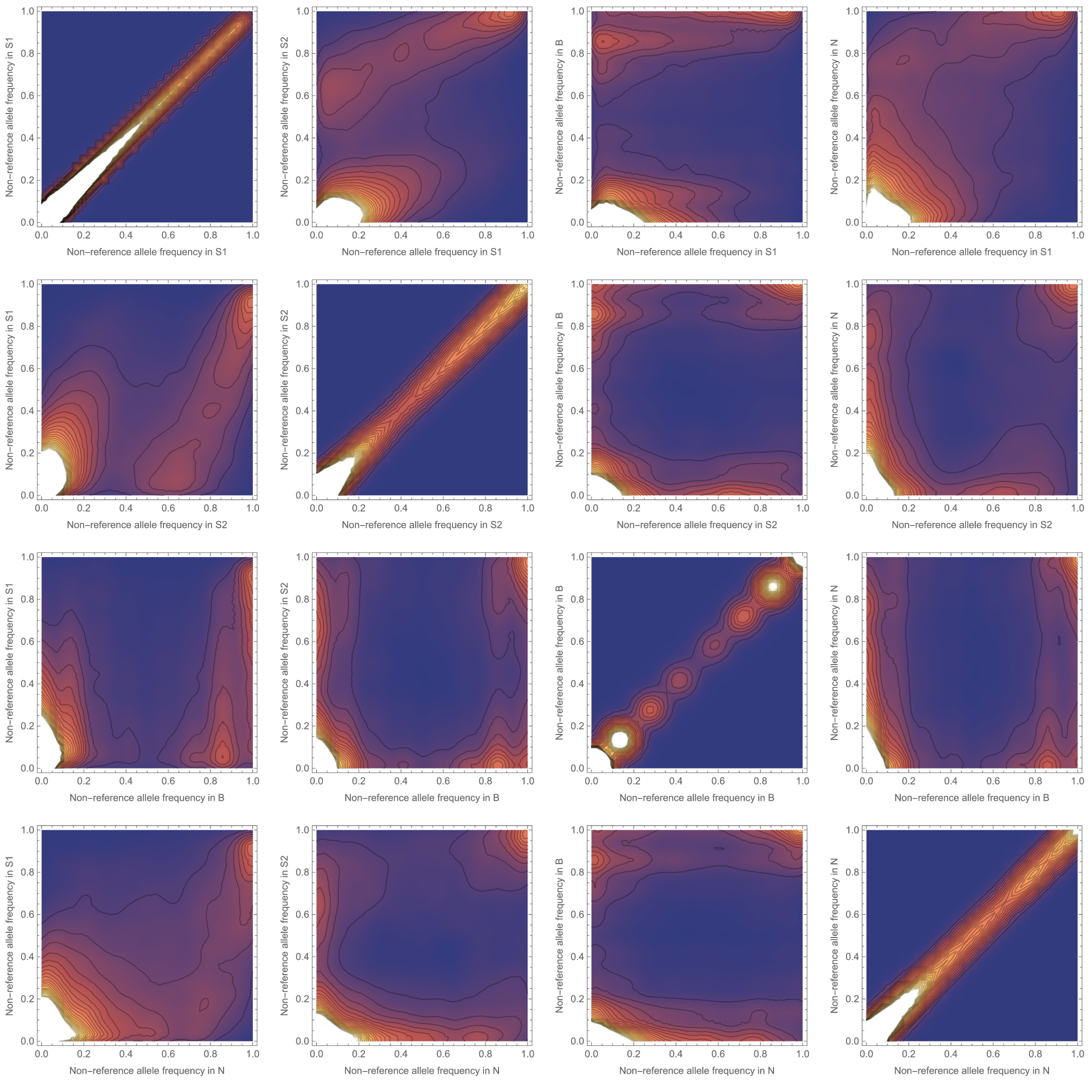

Figure 1—figure supplement 2. Density plot of the joint distribution of the non-reference SNP allele frequencies between the genetic groups. As expected from basic population genetics theory, the non-reference allele tends to be rare in all populations, and the plots have therefore been clipped (white color) to prevent the high density of SNPs close to the origin from obscuring more interesting parts of the distribution.

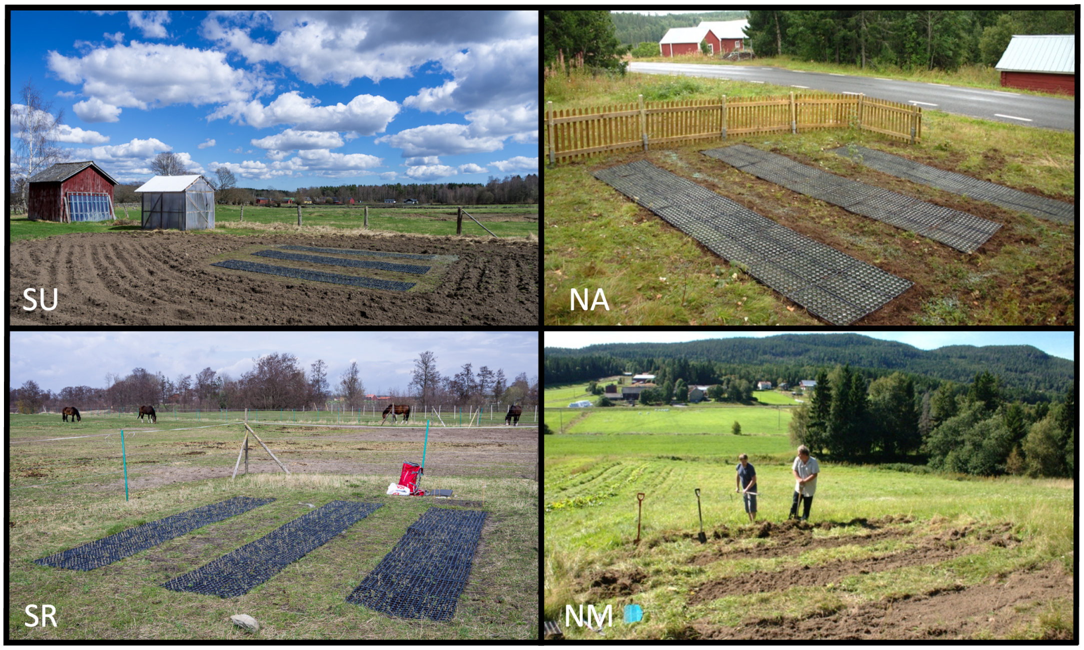

Figure 1—figure supplement 3. Photos of field sites. The four common-garden sites SR and SU in the south, NA and NM in the north. People in NM photo are authors B. B. and S. H.

945

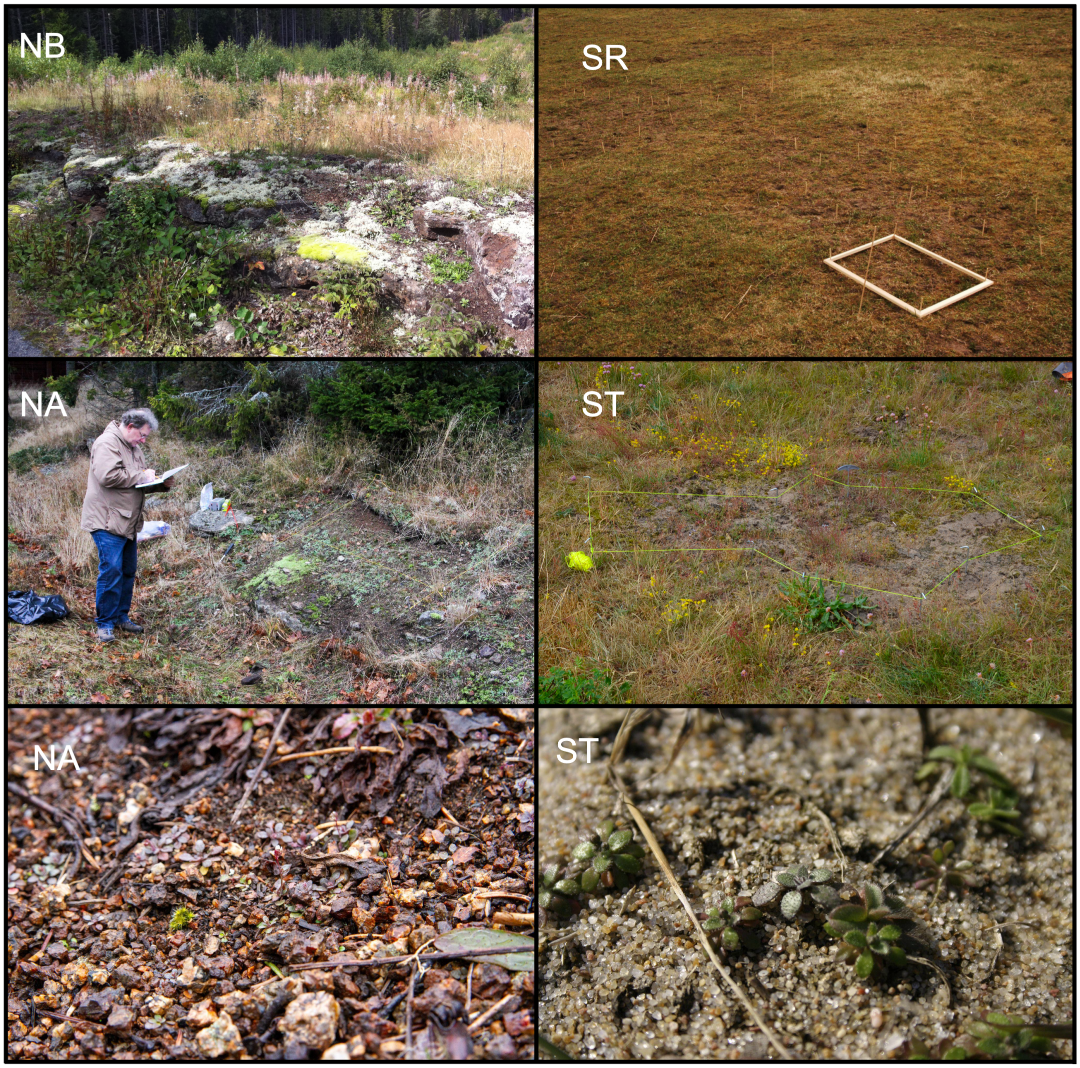

Figure 1—figure supplement 4. Photos of field sites. Plots from each of the four selection experiments: NA, NB in the north and SR and ST in the south. Person shown is author S. H.

946

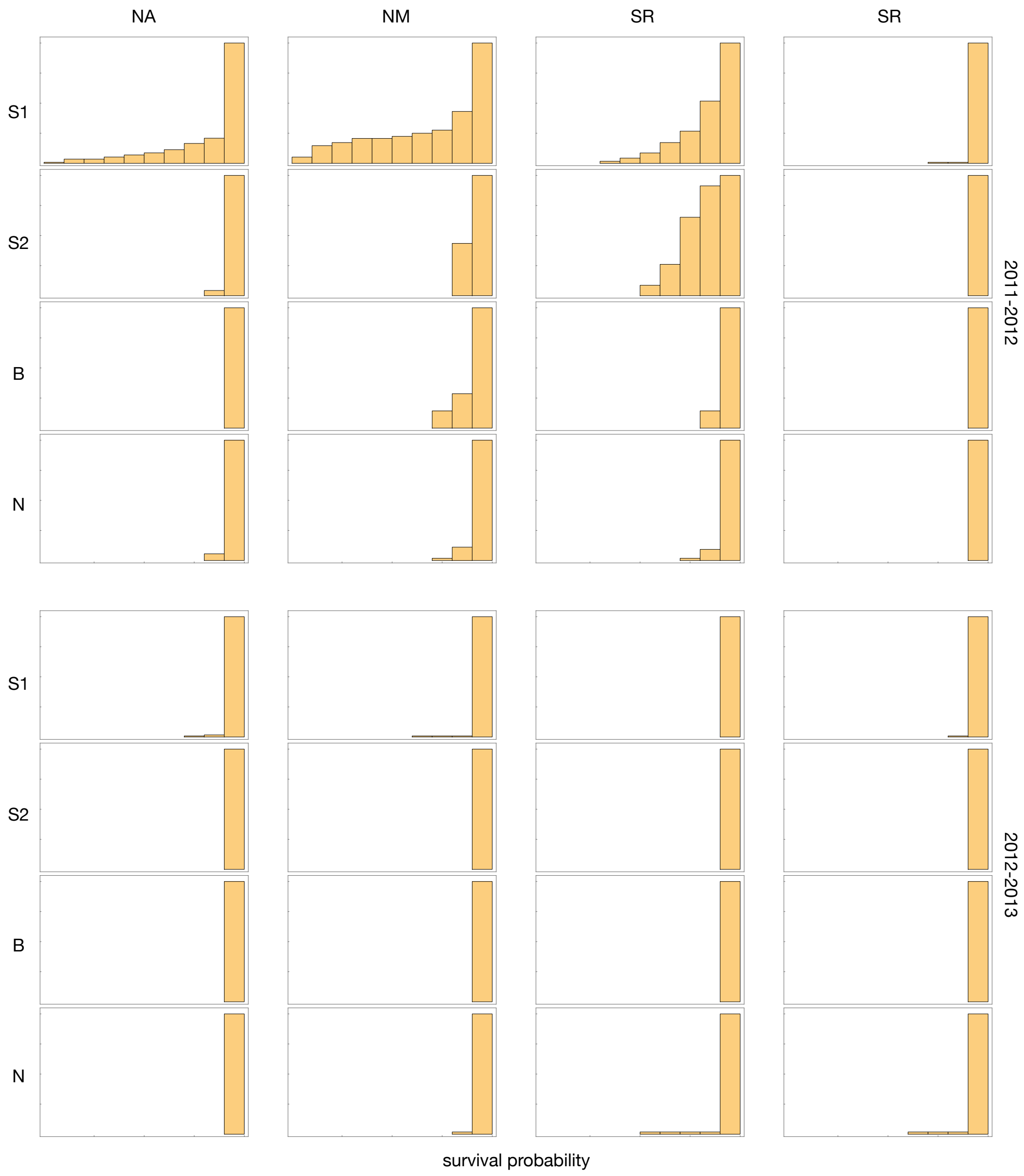

Figure 2—figure supplement 1. Histograms showing the cumulative density of survival estimates across sites and years and groups. In 2011-12, the two northern sites saw high mortality primarily of S1 accessions, and one of the southern sites, SR, saw high mortality primarily of S1 and S2 accession. Mortality in the remaining five experiments was trivial.

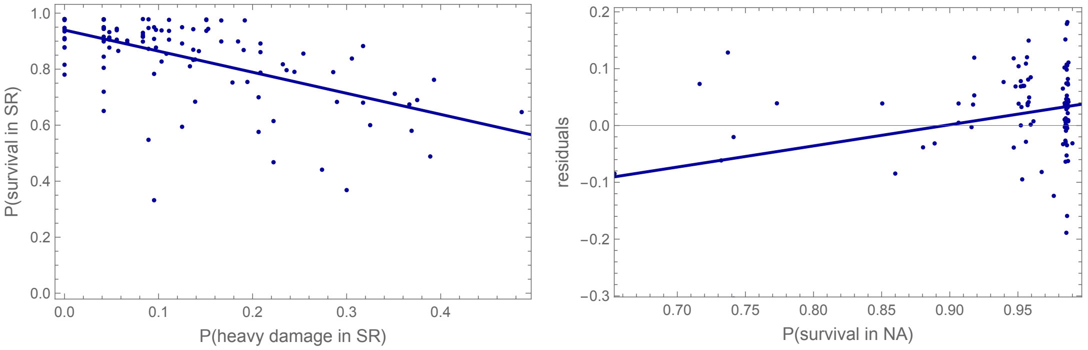

Figure 4—figure supplement 1. Left: Overwinter survival in SR 2011-12 was predicted by slug damage ($R^2_{\text{adj.}} = 0.33; p = 8.6 \times 10^{-12}$). Right: residuals from regression of survival in SR on slug damage remain positively correlated with survival in NA 2011-12, suggesting an underlying shared cause ($R^2_{\text{adj.}} = 0.37; p = 9.3 \times 10^{-14}$).

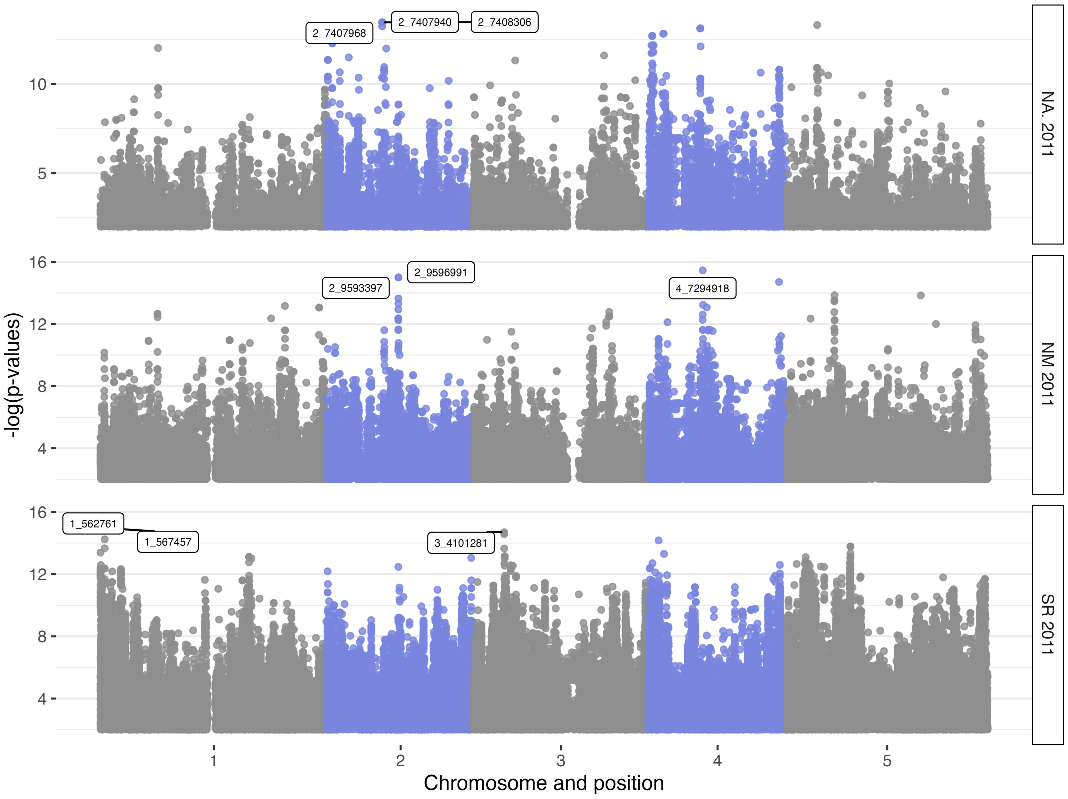

Figure 5—figure supplement 1. Manhattan plots of GWAS for overwinter survival without structure correction.

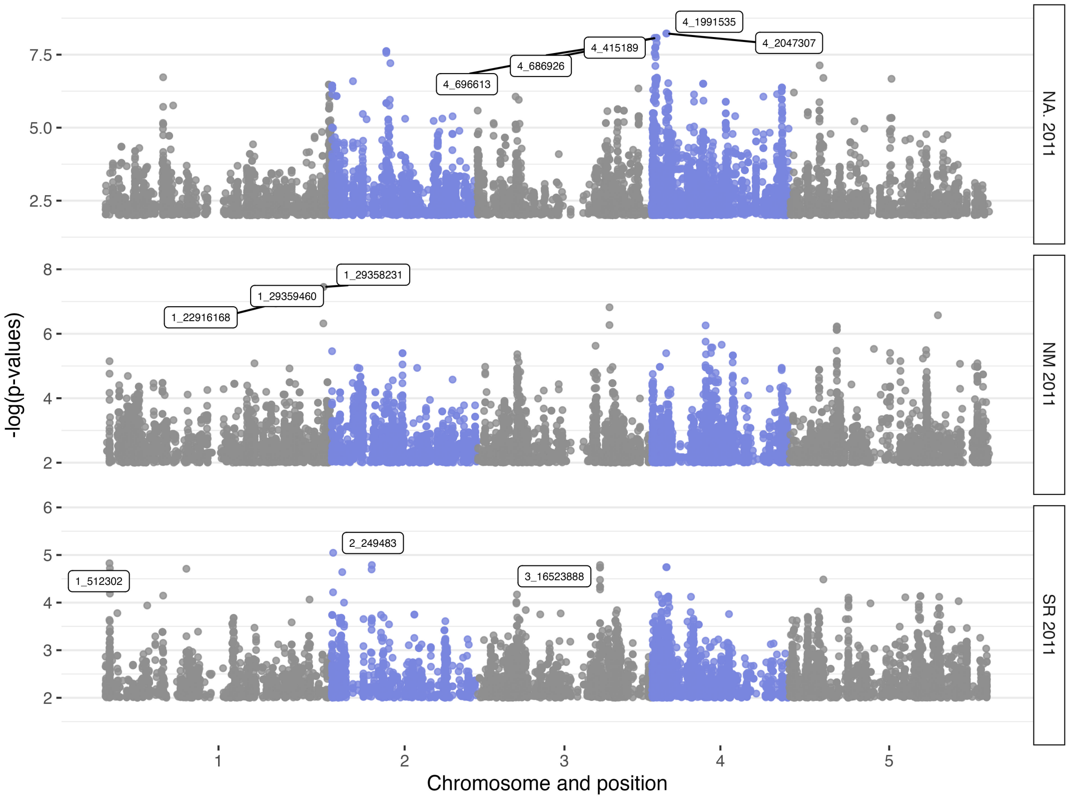

Figure 5—figure supplement 2. Manhattan plots of GWAS for overwinter survival with structure correction.

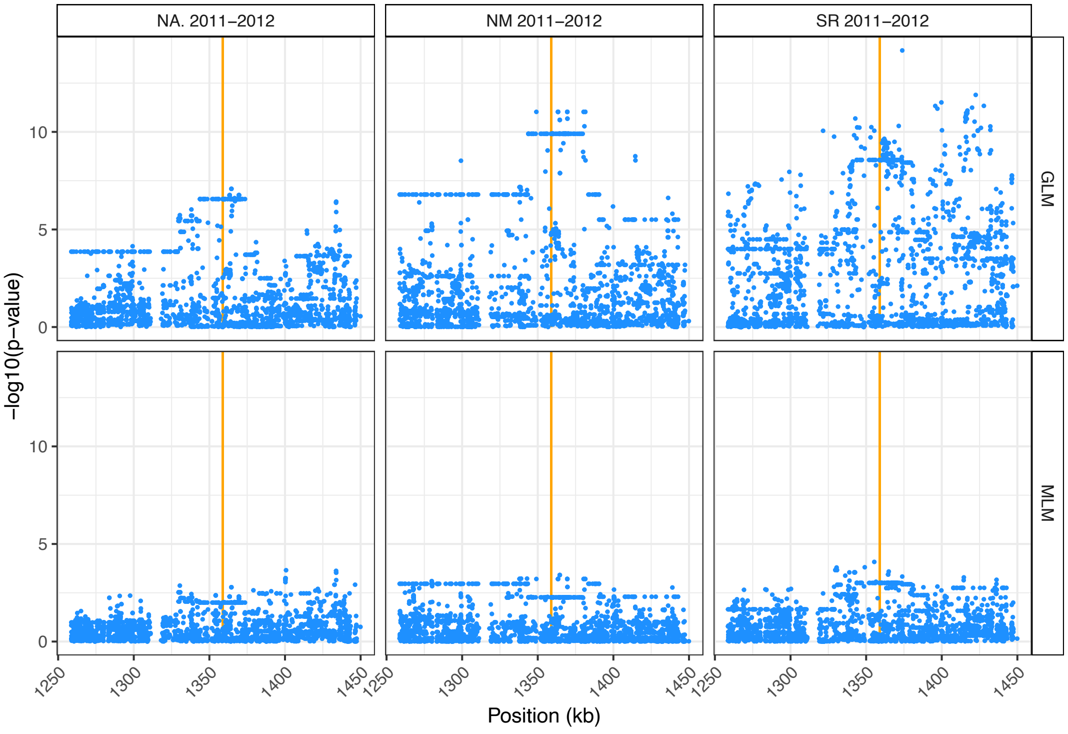

Figure 5—figure supplement 3. Zoom-in on *AOP* region on chromosome 4, without (top row; *cf*. ***Figure 5—figure Supplement 1***) and with (top row; *cf*. ***Figure 5—figure Supplement 2***) structure correction. The orange line shows the location of the gene.

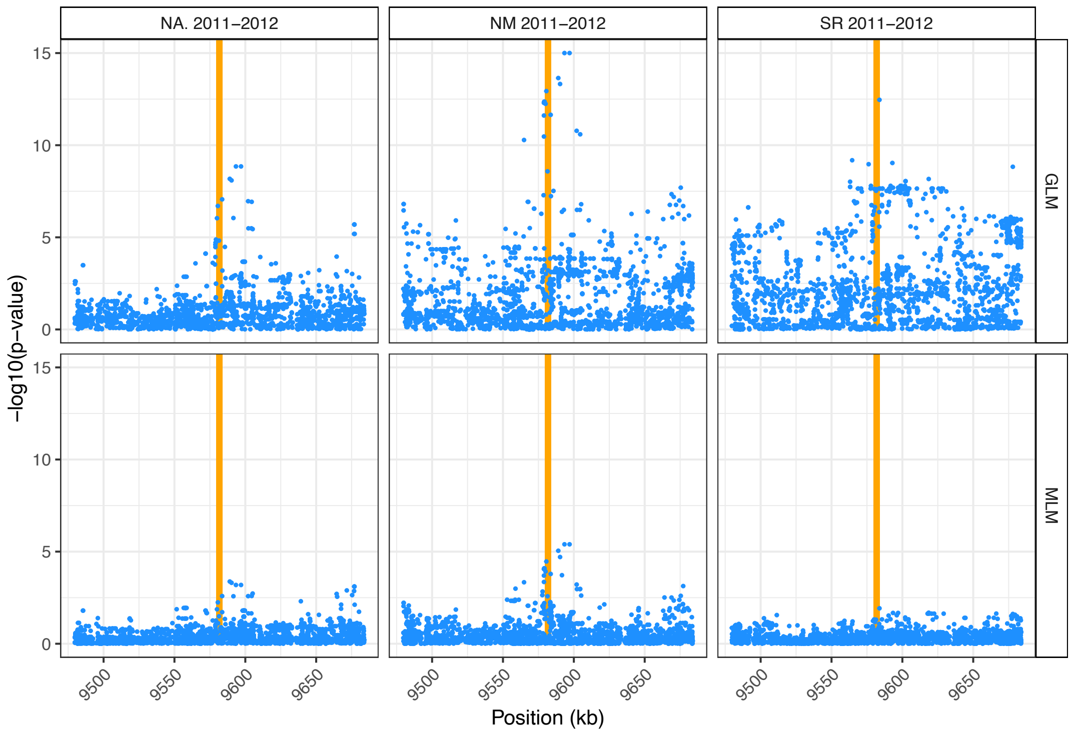

Figure 5—figure supplement 4. Zoom-in on *SVP* region on chromosome 2, without (top row; *cf*. ***Figure 5—figure Supplement 1***) and with (bottom row; *cf*. ***Figure 5—figure Supplement 2***) structure correction. The orange line shows the location of the gene.

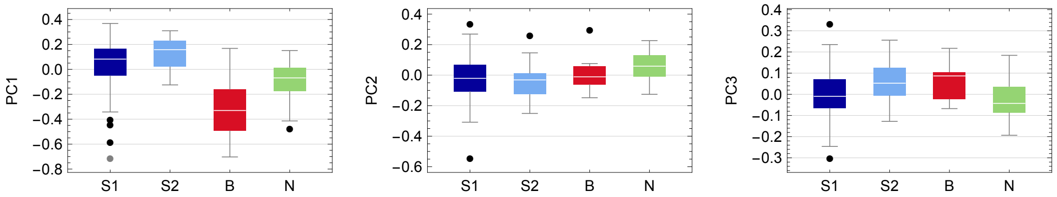

Figure 7—figure supplement 1. Box plots showing the distribution of each PC across accession by group.

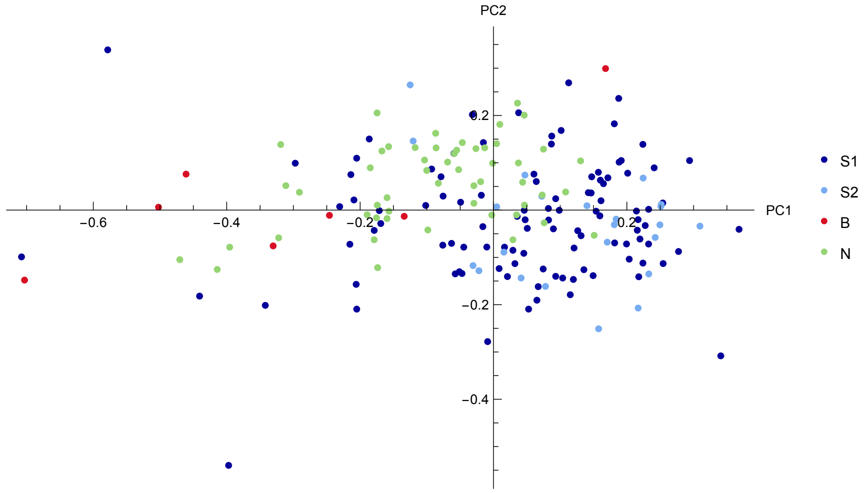

Figure 7—figure supplement 2. Scatter plot showings the accessions, colored by group, in the PC1-PC2 coordinate system.

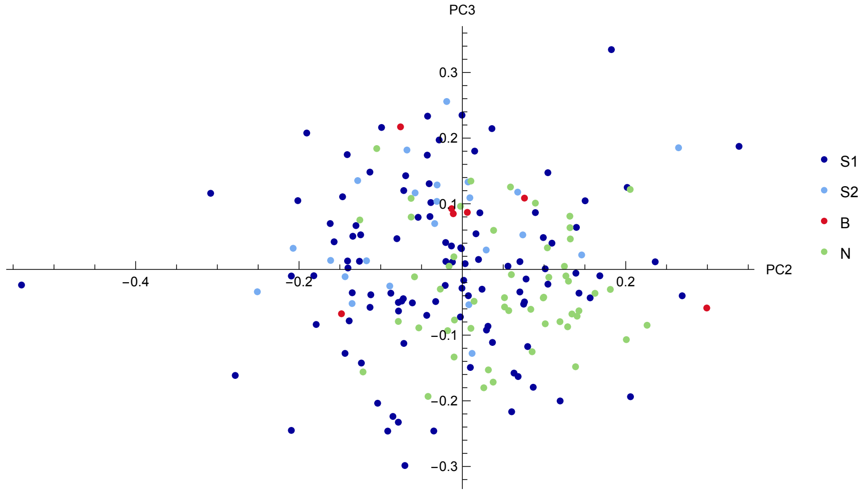

Figure 7—figure supplement 3. Scatter plot showings the accessions, colored by group, in the PC2-PC3 coordinate system.

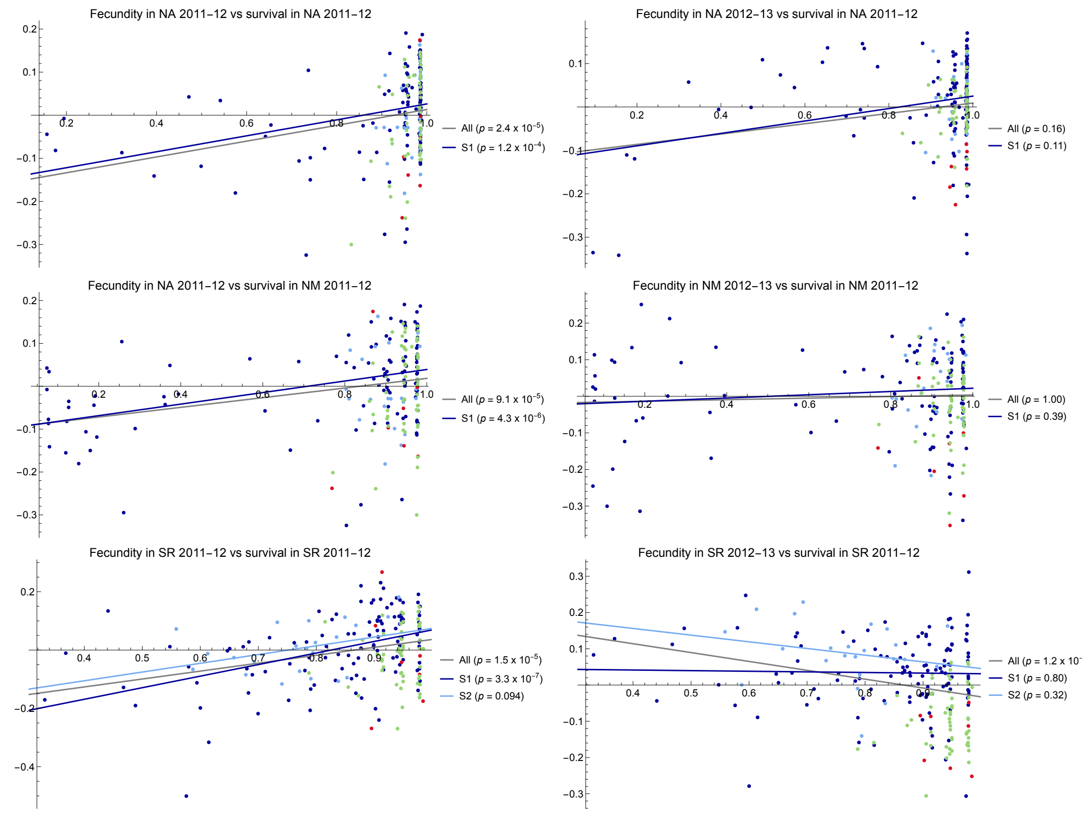

Figure 8—figure supplement 1. The left column shown that overwinter survival and springtime fecundity among survivors is significantly positively correlated in all three experiments with non-trivial mortality, suggesting that a common factor is influencing both. The right column uses fecundity in the following season as a control to demonstrate that no correlation (within groups) exist when no direct causation is possible.

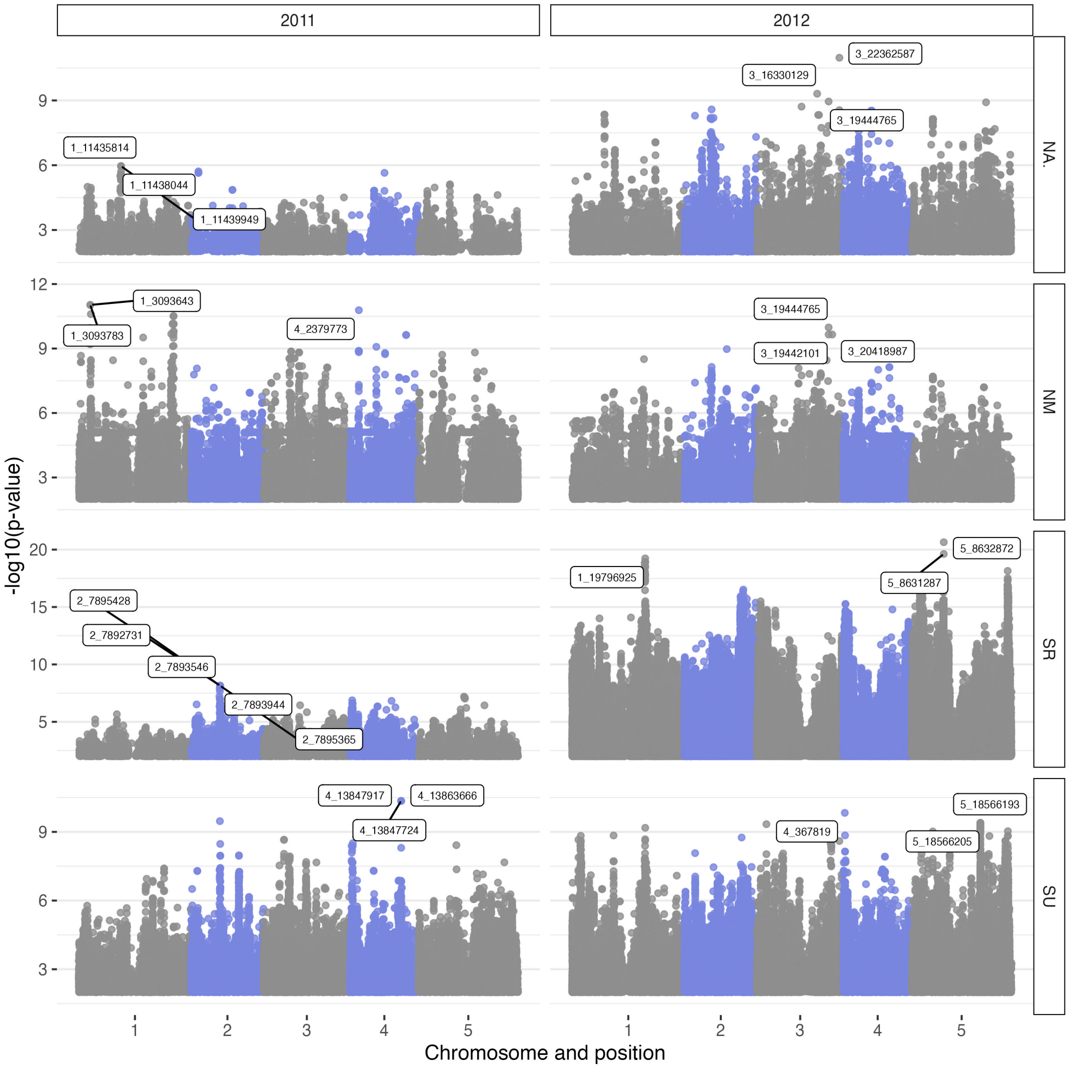

Figure 11—figure supplement 1. Manhattan plots for fecundity GWAS without correction for structure.

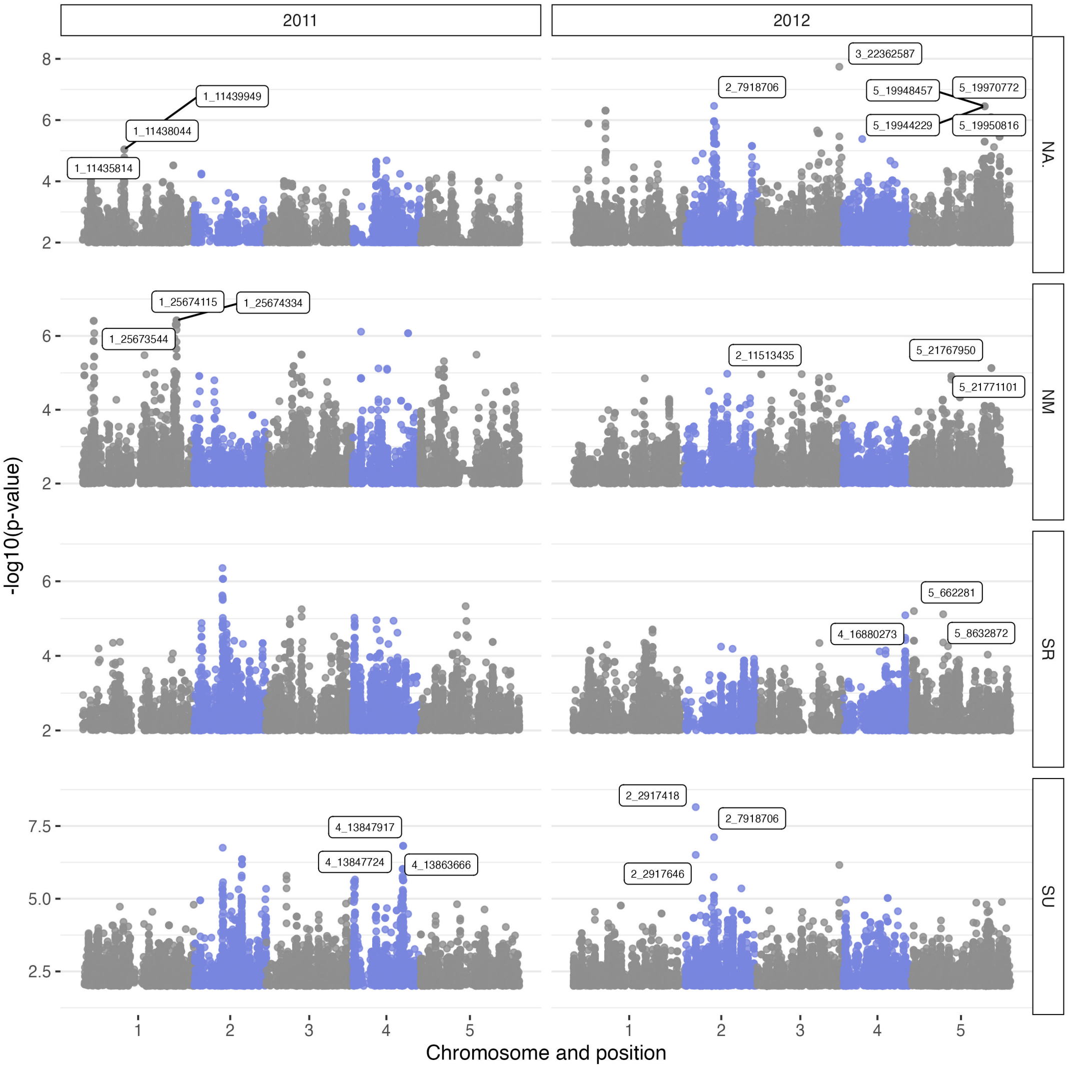

Figure 11—figure supplement 2. Manhattan plots for fecundity GWAS with correction for structure.

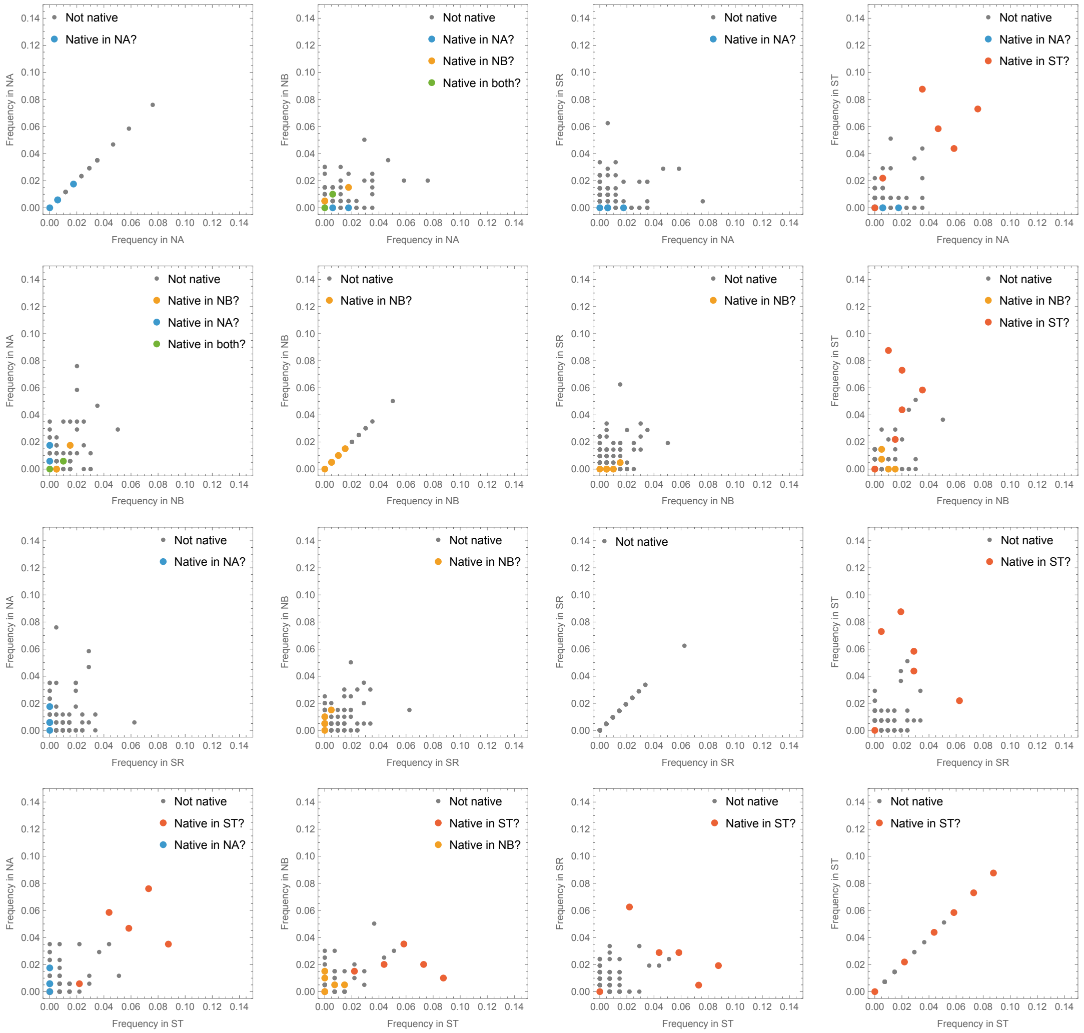

Figure 12—figure supplement 1. Same as ***Figure 12***, but with the two obvious outliers removed to show remaining data better.

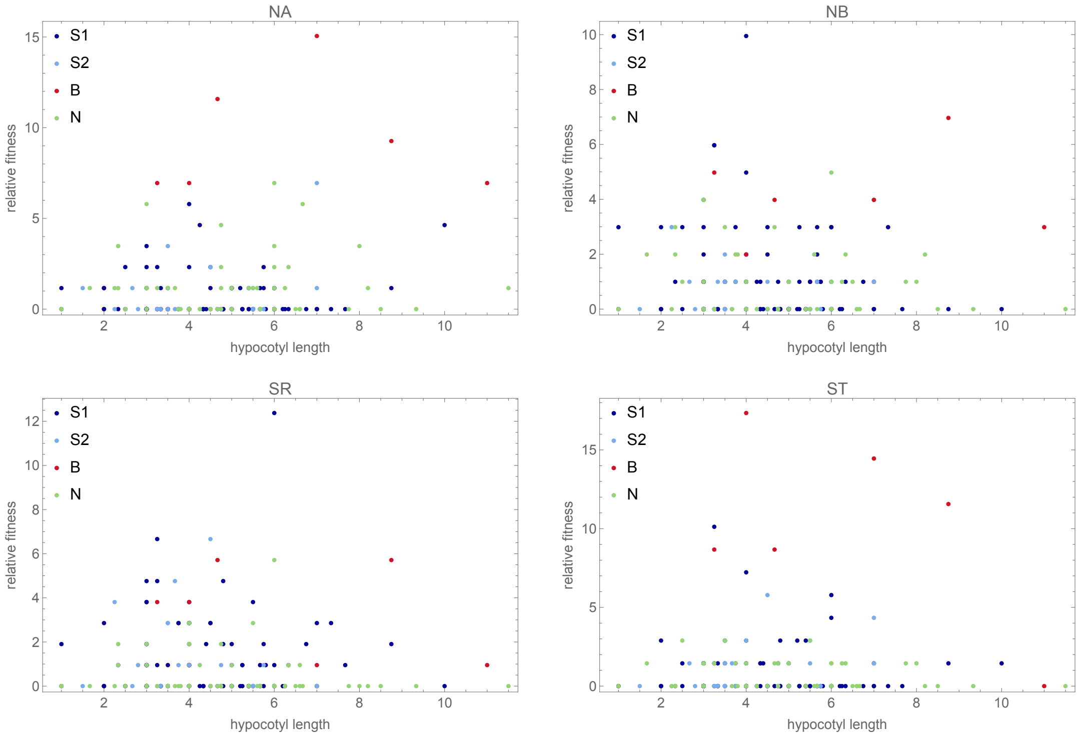

Figure 15—figure supplement 1. Fitness as a function of hypocotyl elongation in each experiment separately.

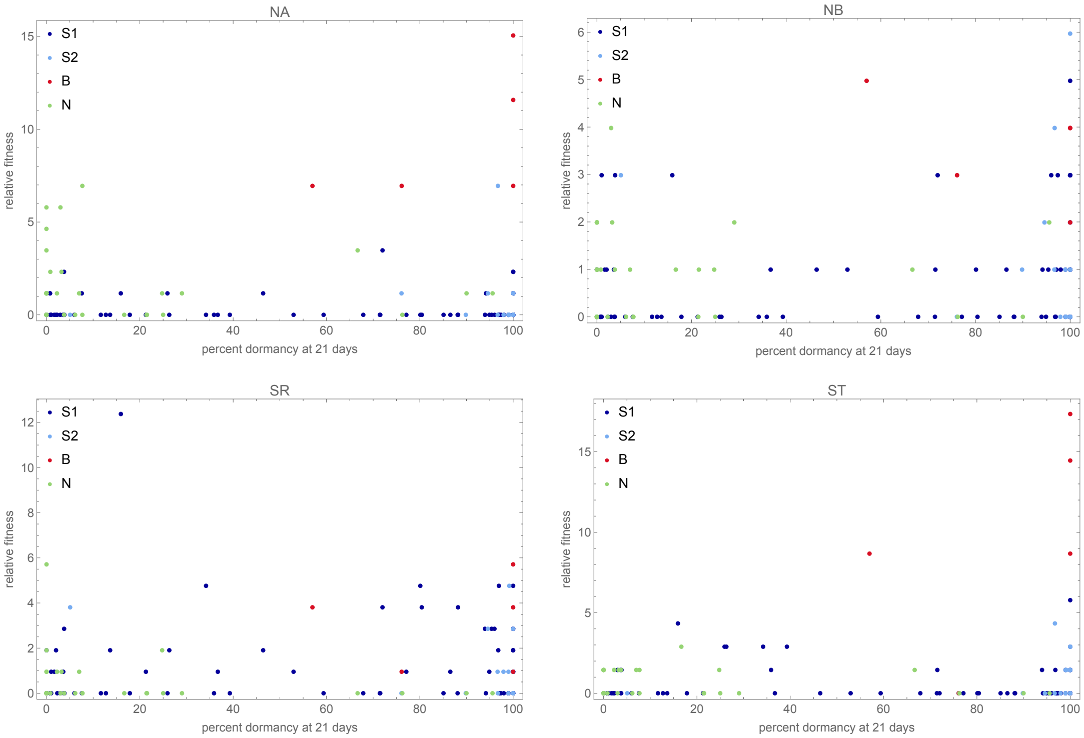

Figure 15—figure supplement 2. Fitness as a function of primary seed dormancy in each experiment separately. High dormancy is necessary (but not sufficient) for high fitness in the southern beach site (ST) but appears to be less important in the other sites, consistent with local adaptation (***Kerdaffrec et al., 2016***).

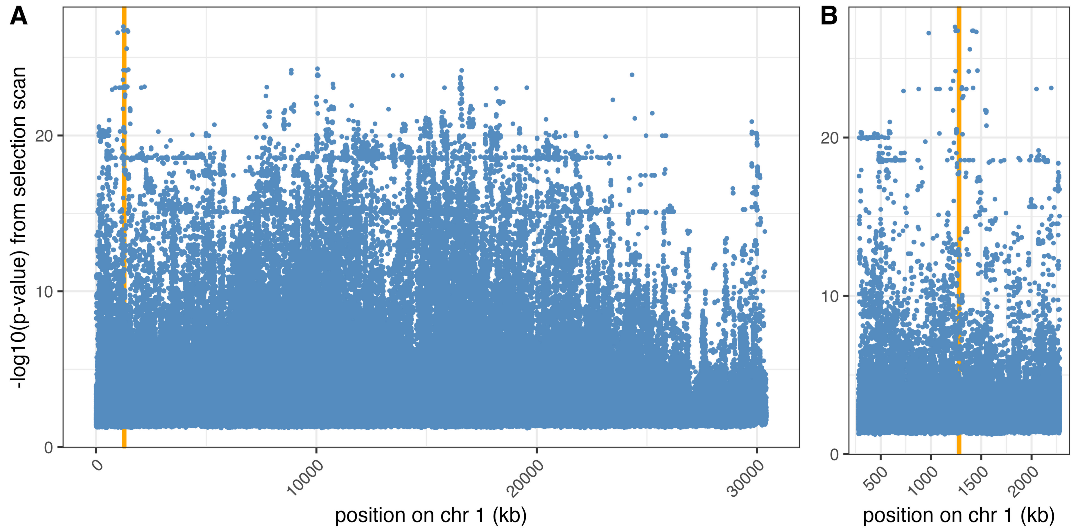

Figure 17—figure supplement 1. Selection scan peak on chromosome 1. Orange vertical lines indicates the position of *YUC3* (AT1G04610) and *MEE4* (AT1G04630). (**A**) Whole chromosome. (**B**) Zoom-in on peak.

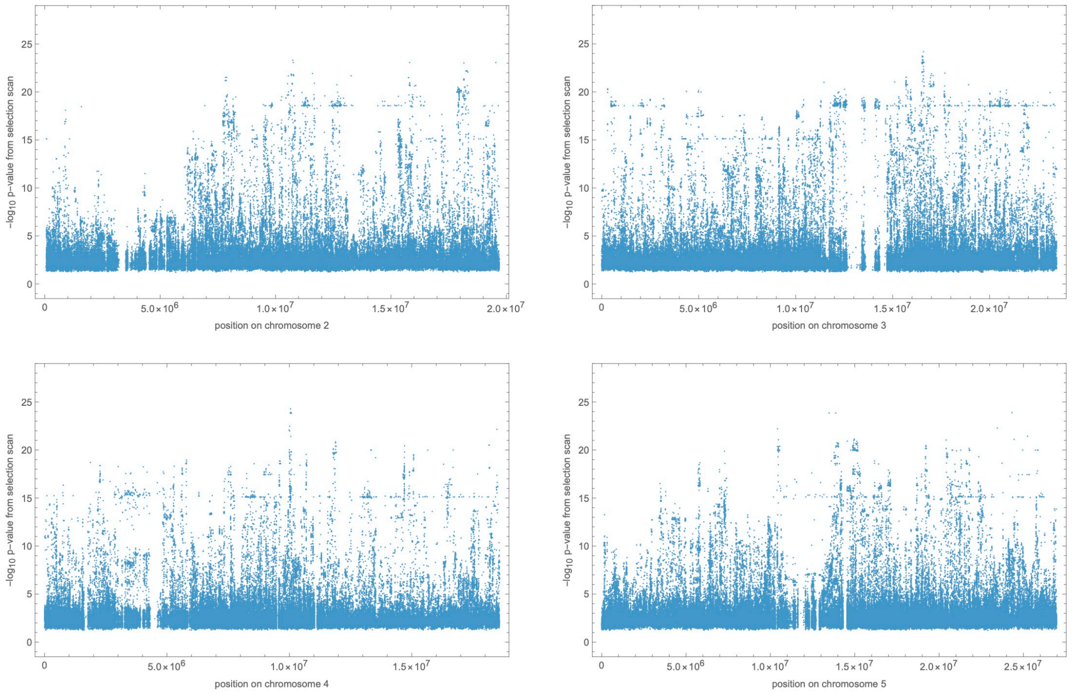

Figure 17—figure supplement 2. Result of selection scan for chromosomes 2–4. Negative log p-values have been summed across the four experiments. For chromosome 1, see ***Figure 17***.

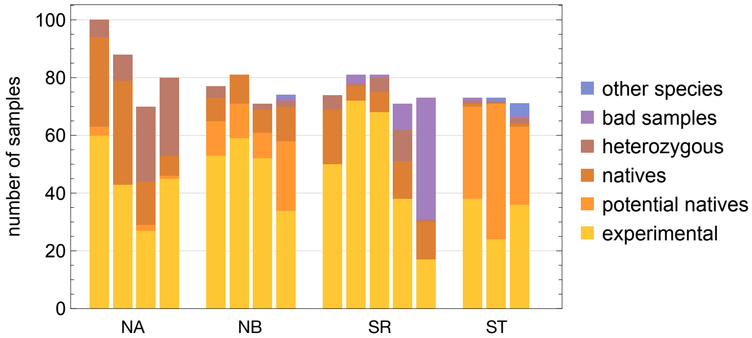

Figure 19—figure supplement 1. The number of samples in each category per site and plot. A minimum of 70 plants were sample per surviving plot.
